# Supplementary material for: Ki-67 is necessary during DNA replication for fork protection and genome stability
Source: Genome Biol. 2024 Apr 22;25:105. doi: 10.1186/s13059-024-03243-5 (PMC11034166; doi:10.1186/s13059-024-03243-5)
Supplement: Supplementary file 1 — Additional file 1: Supplementary Figures. Fig. S1. Generation of AID:mclover endogenously tagged HCT116 cell line. Fig. S2. Ki-67 degradation alters gene expression when cells exit mitosis in its absence. Fig. S3. Ki-67 is in proximity of the replication machinery. Fig. S4. Ki-67 degradation at G1/S leads to CDK2 inactivation. Fig. S5. Correlation between Ki-67 and the interferon pathway. [file 13059_2024_3243_MOESM1_ESM.docx]

**Additional files for**

Ki-67 is necessary during DNA replication for forks protection and genome stability.

Konstantinos Stamatiou ^1^, Florentin Huguet^1^, Lukas V. Serapinas^1^ Christos Spanos^2^, Juri Rappsilber^1,3^ and Paola Vagnarelli^1 *^

**Additional file 1: Fig. S1**

**
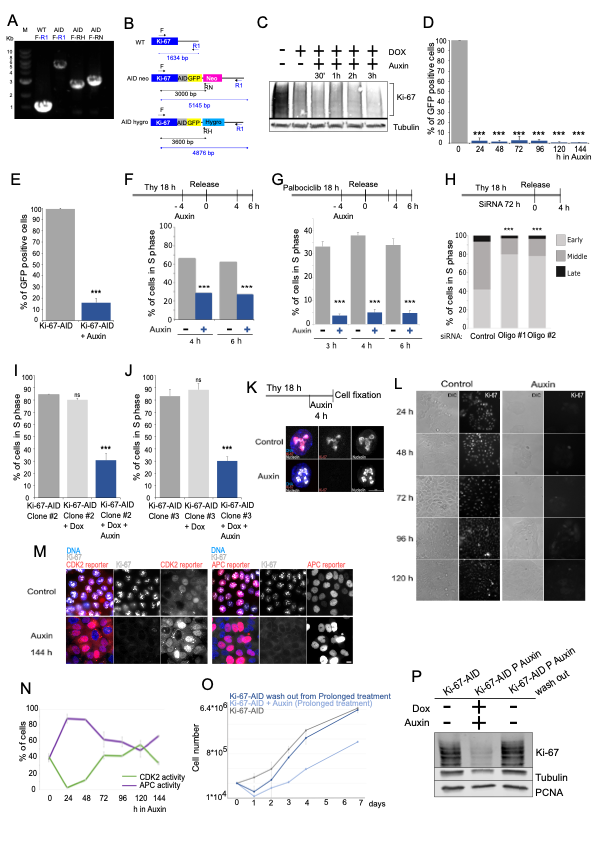
**

**Fig. S1 Generation of AID:mclover endogenously tagged HCT116 cell line.**

A) PCR genotyping of the endogenously tagged AID clones. Gel electrophoresis of the parental cell line (WT) and the targeted cell line (AID) with the indicated primers pairs as shown in (B). M=1kb ladder.

B) Scheme of the expected band sizes obtained by PCR for the wt and targeted alleles with the indicated primers.

C) Western blot of WCL of HCT116:Ki-67-AID untreated treated (-) or treated (+) with Auxin or doxycycline (Dox) for the time indicated. Blots were probed with anti Ki-67 antibody (top) and tubulin (bottom). The lysates were run on an SDS PAGE gradient gel (5-15 %) to resolve all the isoforms of Ki-67. All the isoforms are degraded upon Auxin treatment.

D) Quantification of GFP positive cells in HCT116:Ki-67-AID cell line at 24 to 144 h of Auxin treatment. The graph represents the percentage of GFP positive cells. The values are the average percentage of 3 biological replicas and the error bars represent the standard deviations. Sample size: Control=259; Auxin 24 h=374, 48 h=335, 72 h=330, 96 h=415, 120 h=362 and 144 h=430. The data were statistically analysed with a Chi-squared test (control vs auxin). ***= p<0.001

E) Quantification of GFP positive cells in HCT116:Ki-67-AID cell line at 4 h of Auxin treatment in thymidine. The graph represents the percentage of GFP positive cells. The values are the average percentage of 3 biological replicas and the error bars represent the standard deviations. Sample size: Control=552, Auxin=525. The data were statistically analysed with a Chi-squared test (control vs auxin). ***= p<0.001

F) Top: Scheme of the experiment. BrdU was added 60 minutes before each time point.

Bottom: The graph represents the percentage of BrdU positive cells using the HCT116:Ki-67-AID^CMV:OSTR1^ cell line at the different time points. Sample size: Control 4h=115, 6h=111; Auxin 4h=102, 6h=108. The data were statistically analysed with a Chi-squared test (control vs auxin). ***= p<0.001

G) Top: Scheme of the experiment. EdU was added 30 minutes before each time point.

Bottom: The graph represents the percentage of EdU positive cells at the different time points. The values are the average of 3 biological replicas and the error bars represent the standard deviations. Sample size: Control 3 h=1060, 4 h=1038, 6 h=994; Auxin 3 h=3572, 4 h=3503, 6 h=3444 The data were statistically analysed with a Chi-squared test (control vs auxin). ***= p<0.001

H) Top: Scheme of the experiment. Bottom: Distribution of the replicating cells according to the patterns shown in Figure 1 G. The values are the average of 2 biological replicas and the error bars represent the standard deviations. Sample size: Control=270, Oligo 1#=561, Oligo 2#=291. The data were statistically analysed with a Chi-squared test (control vs auxin). ***= p<0.001.

I and J) HCT116:Ki-67-AID clone #2 (I) and clone #3 (J) cell lines treated with doxycycline and thymidine 24 h and 18 h respectively before the addition of auxin for 4 h. The cells were released from thymidine and EdU was added 30 min before fixation (Fixation time 4 h from thymidine release). The graph represents the percentage of EdU positive cells at 4 h from thymidine release. The values are the average of 2 biological replicas and the error bars represent the standard deviations. Sample sizes: Clone #2 Control=273, Dox= 265 and auxin=337, Clone #3 Control=256, Dox=252 and auxin=331. The data were statistically analysed with a Chi squared test (control vs auxin). ***= p<0.001, ns= not significant.

K) Top: Scheme of the experiment. Thy = Thymidine, Bottom: Representative images of Nucleolin immunostaining using anti Nucleolin antibodies on HCT116:Ki-67-AID cell line without (top) of with (bottom) Auxin. Scale bar 5 μm.

L) Representative images of HCT116:Ki-67-AID Control and Auxin treated cells.

M) Representative images of HCT116:Ki-67-AID-DHB (left) and HCT116: Ki-67-AID-GEMININ (right) of control and 144 h Auxin treatment. The cells were fixed and stained with DAPI (blue). Scale bar 20μm.

N) Quantification of the cell cycle reporter activities in HCT116:Ki-67-AID-DHB and HCT116:Ki-67-AID-GEMININ cell lines. Green: HCT116:Ki-67-AID-DHB, Sample size: Control=305, Auxin: 24 h=214, 48 h=226, 72 h=253, 96 h=254, 120 h=212, 144 h=215. Purple: HCT116:Ki-67-AID-GEMININ, Sample size: Control=333, Auxin: 24 h=289, 48 h=310, 72 h=286, 96 h=342, 120 h=332, 144 h=404. The values represent the average of 2 independent experiments and the error bars are the standard deviations.

O) Growth curves of the degron tagged HCT116:Ki-67-AID (Ki-67-AID), HCT116:Ki-67-AID (+ Auxin) and Auxin prolonged treatment and HCT116:Ki-67-AID wash out from Auxin prolonged treatment. The values represent the average of 2 independent experiments. The error bars represent the standard deviations.

P) Western blot of Whole Cell Lysate of HCT116:Ki-67-AID (Ki-67-AID), HCT116:Ki-67-AID (+ Auxin)- Auxin prolonged treatment - and HCT116:Ki-67-AID wash out from Auxin prolonged treatment. Blots were probed with anti Ki-67 antibody (top),tubulin (middle) and PCNA (bottom). The lysates were run on an SDS PAGE gradient gel (5-15 %) to resolve all the isoforms of Ki-67. All the isoforms are degraded upon Auxin treatment.

**Additional file 1: Fig. S2**

**Fig. S2 Ki-67 degradation alters gene expression when cells exit mitosis in its absence.**

A) Volcano plot of the differentially expressed genes when Ki-67 is degraded in cells arrested in nocodazole and then released in Thymidine with or without Auxin for 18 h. The pink line represents p-value < 10e^-20^, the Hot pink line p-value=10e^-20^-10e^-30^ and the purple line p-value=10e^-20^-10e^-60^. Several genes are differentially expressed in this sample. B) Table of DNA replication genes downregulated in G1 upon Ki-67 degradation in mitosis. C) Venn diagram comparing the numbers of upregulated and downregulated genes in the RNA-seq experiment in (A) (Mitosis-G1) and in Figure 3 A (G1). D) Magic analyses of upregulated genes using a matrix containing the gene bodies (from the promoter to the end of the last exon and 1kb flanking sequence either side of the gene body). E-I) Western blots of whole cell lysate of the indicated cell lines upon Control or Ki-67 RNAi. Oligo #1 and Oligo #2 indicate two already published SiRNA oligos. The blots were probed with anti Ki-67 (top panels) and anti alpha tubulin (bottom panels) antibodies. The graphs show the quantification of the blots.

**Additional file 1: Fig. S3**

**Fig. S3- Ki-67 is in proximity of the replication machinery**

A) PCR genotyping of the endogenously tagged APEX2 clones. Gel electrophoresis of the parental cell line (WT) and the targeted cell line (APEX2) with the indicated primers pairs as shown in (B). M=1kb ladder

B) Scheme of the expected band size obtained by PCR for the wt and targeted alleles with the indicated primers.

C) Violin plot of the quantification of the experiment in (D). The box inside the violin represents the 75th and 25th percentile, whiskers are the upper and lower adjacent values, and the line is the median. Sample size: Control=53, Auxin=74. A Wilcoxon test was conducted for comparing the experiments and *** = p<0.001.

D) Representative images of the proximity ligation assay (PLA) using anti MCM3 and anti GFP antibodies on HCT116:Ki-67-AID^CMV:OSTR1^ cell line without (left) of with (right) Auxin. Scale bar 15 μm.

E) Line plots (right) of the nucleus of HCT116:Ki-67-AID across the yellow lines shown in the images (left panels). Scale bar 5 μm.

F) Line plots of Ki-67 foci and BrdU foci across the white lines shown in the images (left panels). Scale bar 5 μm.

**Additional file 1: Fig. S4**

**Fig. S4 - Ki-67 degradation at G1/S leads to CDK2 inactivation.**

A) Scheme of the experiment in (B). Thy = Thymidine B) (Top) Representative Western blot of HCT116: Ki-67-AID cell line blocked with Thymidine then untreated (Ki-67 AID) or treated with Auxin for 4h (Ki-67 AID Auxin) in the presence (+) or absence (-) of BI8626. The blots were probed with anti GAPDH and anti CHK1 (B) antibodies. The graphs at the top represent the quantification of the blots. The values represent the average of 3 independent replicas and the error bars are the standard deviations. The experiments were analysed by a Student’s t-test. *=p<0.05, ***= p< 0.001.

C) Scheme of the experiment (top) and representative images of the DHB-mCherry reporter localisation in cells at the G1/S boundary with or without Ki-67 (bottom). Thy = Thymidine. Scale bar 10 μm.

D) Quantification of the experiment in (C). The violin plots represent the distribution of the cytoplasmic/nuclear mean signals of 3 biological replicates. The box inside the violin represents the 75^th^ and 25^th^ percentile, whiskers are the upper and lower adjacent values and the line is the median. N: Control=336 Auxin=322. The data were analysed with a Wilcoxon test. ***=p<0.001.

**Additional file 1: Fig. S5**

**Fig. S5 - Correlation between Ki-67 and the interferon pathway.**

A) Scheme of the expected band size obtained by PCR for the wt and targeted alleles with the indicated primers.

B) PCR genotyping of the Ki-67-AID-STING-KO cell line. The black and blue arrows indicate the wt and the KO alleles respectively (as shown in A). M =kb ladder.

C and D) R2 genomics analyses of patient and experimental data sets for IFIT1 (C) and STAT2 (D) compared against MKI67 gene expression, X and Y-axis the log2 transformed average expression level and the standard deviation is represented.

E) R2 genomics analyses of patient and experimental data sets for IFIT1 compared against MYC gene expression, X and Y-axis the log2 transformed average expression level and the standard deviation is represented.

F-G) Western blots of whole cell lysate of HCT116:Ki-67-AID and HCT116:Ki-67-AID^CMV:OSTR1^ (F), HCT116: Ki-67-AID untreated (control) and HCT116: Ki-67-AID maintained always in Dox and Auxin (G). The blots were probed with anti Ki-67 (1^st^ panel), anti alpha tubulin (2^nd^ panel) and PCNA (3^rd^ panel) antibodies.

H) HCT116:Ki-67-AID, HCT116:Ki-67-AID^CMV:OSTR1^ and HCT116:Ki-67-AID cells were transfected with a plasmid carrying the luciferase gene under the control of the Interferon β promoter together with a plasmid carrying renilla. The graph represents the luciferase activation normalised to renilla in cells HCT116:Ki-67-AID^TET-ON:OSTR1^, HCT116:Ki-67-AID^CMV:OSTR1^ and HCT116:Ki-67-AID maintained always in Dox and Auxin. The values represent the average of 3 independent experiments for HCT116:Ki-67-AID and HCT116:Ki-67-AID maintained always in Dox and Auxin and the average of 2 independent experiments for HCT116:Ki-67-AID, HCT116:Ki-67-AID^CMV:OSTR1^ the error bars are the standard deviations. The experiments were analysed by a Student’s t-test. ***=p<0.001

I) Correlation of Relative luciferase expression from (H) and relative Ki-67 protein level from (F-G).

J) Correlation of Relative luciferase expression from (H) and the doubling time (DT) of the 3 cell lines (F-G). DT indicates the doubling time in h.
