# Supplementary material for: Ki-67 is necessary during DNA replication for fork protection and genome stability
Source: Genome Biol. 2024 Apr 22;25:105. doi: 10.1186/s13059-024-03243-5 (PMC11034166; doi:10.1186/s13059-024-03243-5)
Supplement: Supplementary file 2 — Additional file 2: Supplementary Tables. Table S1. Oligonucleotides used in this study. Table S2. List of Antibodies, reagents and cell lines in this study. [file 13059_2024_3243_MOESM2_ESM.docx]

Table 1: Oligonucleotides used in this study

| **Control RNAi:**  5′- CGUACGCGGAAUACUUCGA-3′ | [1] |
| --- | --- |
| **Ki-67 RNAi Oligo 1:**  5′- GCAUUUAAGCAACCUGCAA-3′ | [2] |
| **Ki-67 RNAi Oligo 2:**  5′- CGUCGUGUCUCAAGAUCUATT-3′ | [3] |
| **gRNA Pair 1**  5′- CACCGTTCACTGTCCCTATGACTTC-3′ | This paper |
| **gRNA Pair 2**  CACCGATTTGACAGAAAAATCGAAC | This paper |
| **gRNA Pair 3**  CACCGTTTGACAGAAAAATCGAACT | This paper |
| **Ki-67 HA plasmid sequence investigation Forward:**  5′- CACATGCTCCTCTTTGCCGA-3′ | This paper |
| **Ki-67 HA plasmid sequence investigation Reverse:**  5′-GCCACGAGTCACTCCTTACA-3′ | This paper |
| **Cell lines genotyping Ki-67 Forward:**  5′- AAGCAGAAGACACCCGACAG-3′ | This paper |
| **Cell lines genotyping Ki-67 Reverse:**  5′- TCCCAAGAGACCAAGGCAAG-3′ | This paper |
| **Cell lines genotyping Neomycin Reverse**  5′- GCCCAGTCATAGCCGAATAG-3′ | This paper |
| **Cell lines genotyping Hygromycin Reverse**  5′- AGACGCTGTCGAACTTTTCG-3′ | This paper |
| **STING KO Cell line genotyping STING Forward**  5′- GAAGTGCCCAGCCAGAGC -3′ | Laboratory of Prof. Frank van Kuppeveld (Utrecht University, Netherlands) |
| **STING KO Cell line genotyping STING Reverse**  5′- CACAGTCCTCCAGTAGCTGC-3′ | Laboratory of Prof. Frank van Kuppeveld (Utrecht University, Netherlands) |

Table 2: List of Antibodies, reagents and cell lines in this study

| **REAGENT or RESOURCE** | **SOURCE** | **IDENTIFIER** |
| --- | --- | --- |
| **Antibodies** | | |
| Mouse monoclonal anti-MCM3(clone E-8) | Santa Cruz Biotechnology | Cat# Sc-390480 |
| Mouse monoclonal anti-PCNA (clone PC10) | Santa Cruz Biotechnology | Cat# Sc-56  RRID:AB_628110 |
| Mouse monoclonal anti-ORC1 (clone F-10) | Santa Cruz Biotechnology | Cat# Sc-398734 |
| Mouse monoclonal anti-p21 Waf1:Cip1 (clone F-5) | Santa Cruz Biotechnology | Cat# Sc-6246  RRID:AB_628073 |
| Mouse monoclonal anti-p53 (clone DO-1) | SIGMA | Gift from Dr. Evgeny Makarov |
| Mouse monoclonal anti-c-Myc (clone C-33) | Santa Cruz Biotechnology | Cat#Sc-42  RRID:AB_2282408 |
| Mouse monoclonal anti-a-tubulin (clone B-5-1-2) | Sigma-Aldrich | Cat# T5168  RRID:AB_477579 |
| Mouse monoclonal anti-GAPDH | Proteintech | Cat# 60004-1-Ig, RRID:AB_2107436 |
| Rabbit polyclonal anti GFP [PABG1] | Chromotek | Cat# PABG1-20, RRID:AB_2749857 |
| Mouse monoclonal anti-BrdU Alexa Fluor® 647 (clone 3d4) | Biolegent | Cat# 364108  RRID:AB_2566452 |
| Mouse monoclonal anti-Ki-67 | BD Biosciences | Cat# 610968  RRID:AB_398281 |
| Rabbit polyclonal anti-Nucleolin | Abcam | Cat# 22758  RRID:AB_776878 |
| Rabbit monoclonal anti-Histone H2A.X (clone D17A3) | Cell signalling | Cat# 7631  RRID:AB_10860771 |
| Mouse monoclonal anti-p-Histone H2A.X Antibody (Ser 139) | Santa Cruz Biotechnology | Cat# Sc-517348  RRID:AB_2783871 |
| Rabbit polyclonal anti-Histone H3 | Active Motif | Cat# 39163  RRID:AB_2614978 |
| Mouse monoclonal anti-H3K27me2/3 | Active Motif | Cat# 39535  RRID:AB_2793246 |
| Rabbit polyclonal anti-H3K9me3 | Active Motif | Cat# 39161  RRID:AB_2532132 |
| Mouse monoclonal anti-H4K20me1 | Active Motif | Cat# 39727  RRID:AB_2615074 |
| Rabbit polyclonal anti-H3K9me2 | Active Motif | Cat# 39239  RRID:AB_2793199 |
| Mouse polyclonal Texas Red | Jackson Immunoresearch | Cat# 715-585-150  RRID:AB_2340854 |
| Goat polyclonal anti-Mouse HRP | Thermo Fisher Scientific | Cat# 31444  RRID:AB_228321 |
| Goat polyclonal anti-Mouse 800CW | LI-COR | Cat# 926-32210  RRID:AB_621842 |
| Goat polyclonal anti-Rabbit 680RD | LI-COR | Cat# 926-68071  RRID:AB_10956166 |
| **Chemicals, peptides, and recombinant proteins** | | |
| 3-Indoleacetic acid | Sigma-Aldrich | Cat# I2886  CAS:87-51-4 |
| Thymidine | Sigma-Aldrich | Cat# T9250  CAS:50-89-5 |
| RO-3306 | Sigma-Aldrich | Cat# SML0569  CAS:872573-93-8 |
| Nocodazole | Sigma-Aldrich | Cat# M1404  CAS:31430-18-9 |
| MG132 | Millipore | Cat# 474787  CAS:133407-82-6 |
| Doxycycline | Sigma | Cat# D9891  CAS: 24390-14-5 |
| Palbociclib (PD-0332991) | Selleckchem | Cat# S1116  CAS: 827022-32-2 |
| HUWE1 inhibitor BI8626 | MedChemExpress | Cat# HY-120204  CAS: 1875036-75-1 |
| **Critical commercial assays** | | |
| Click-iT™ EdU Cell Proliferation Kit for Imaging, Alexa Fluor™ 647 | Thermo Fisher Scientific | Cat# C10340 |
| Dual-Luciferase Reporter Kit | Promega | Cat# E1910 |
| Duolink® In Situ PLA® Probe Anti-Rabbit MINUS | Sigma-Aldrich | Cat# DUO92005 |
| Duolink® In Situ PLA® Probe Anti-Mouse PLUS | Sigma-Aldrich | Cat# DUO92001 |
| Duolink® In Situ Detection Reagents Red | Sigma-Aldrich | Cat# DUO92008 |
| Monarch Total RNA Miniprep Kit | New England BioLabs | Cat# T2010S |
| JetPrime® | Polyplus transfection® | Cat# 101000015 |
| Nick Translation Kit | Abbott | Cat# 07J00-001 |
| pGEM-T Easy empty vector | Promega | Cat# A1360 |
| **Deposited data** | | |
| RNA sequencing data | This paper | E-MTAB-12279 |
| Proteomics data | This paper | PXD037513 |
| Human reference genome GRCh38 | Genome Reference Consortium Human Build 38 | https://www.ncbi.nlm.nih.gov/assembly/GCF_000001405.26/ |
| **Experimental models: Cell lines** | | |
| HCT116(wt) | Laboratory of Dr Anabelle Lewis (Brunel University of London, UK) | ATCC, CCL-247 |
| HCT116 (wt, MLH1 rescued) | Laboratory of Dr Anabelle Lewis (Brunel University of London, UK) | Horizon Discovery |
| SW480 | Laboratory of Dr Anabelle Lewis (Brunel University of London, UK) | ATCC, CCL-228 |
| hTERT-RPE1 | Laboratory of Dr Viji Draviam (Queen Marry University of London, UK) | ATCC, CRL-4000 |
| HCT116 TET-ON:OSTR1 | Laboratory of Dr Masato T. Kanemaki (University of Tokyo, Japan) | N/A |
| HCT116:Ki-67-AID CMV:OsTR1 | Laboratory of Dr Masatoshi Takagi (RIKEN Institute, Japan) | N/A |
| HCT116:Ki-67-AID | This paper | N/A |
| HCT116:Ki-67-AID mCherry-DHB | This paper | N/A |
| HCT116:Ki-67-AID mCherry-GEMININ | This paper | N/A |
| HCT116:Ki-67-APEX2 | This paper | N/A |
| HCT116:Ki-67-AID STING-KO | This paper | N/A |
| HeLa Kyoto | ATCC | CRL-11268 |
| **Oligonucleotides** | | |
| See Table S1 for Oligonucleotides used in this study |  |  |
| **Recombinant DNA** | | |
| pX330-U6-Chimeric_BB-CBh-hSpCas9 | [4] | Addgene plasmid #42230 |
| pMK289 (mAID-mClover-NeoR) | [5] | Addgene plasmid #72827 |
| pMK290 (mAID-mClover-Hygro) | [5] | Addgene plasmid #72828 |
| APEX2-csChBP | [6] | Addgene plasmid # 108876 |
| pBluescript II SK(+)-Ki-67 | This paper | Biomatik |
| pBluescript II SK(+)-Ki-67 - mAID-mClover-Neo | This paper | N/A |
| pBluescript II SK(+)-Ki-67 - mAID-mClover-Hygro. | This paper | N/A |
| pX330-U6-Chimeric-BB-CBh-hSpCas9-gRNA-P1 | This paper | N/A |
| pX330-U6-Chimeric-BB-CBh-hSpCas9-gRNA-P2 | This paper | N/A |
| pX330-U6-Chimeric-BB-CBh-hSpCas9-gRNA-P3 | This paper | N/A |
| pBluescript II SK(+)-Ki-67-APEX2-mClover-Neo | This paper | N/A |
| pBluescript II SK(+)-Ki-67-APEX2-mClover-Hygro | This paper | N/A |
| pCRISPR-hCas-2xSTING-Puro | Laboratory of Prof. Frank van Kuppeveld (Utrecht University, Netherlands) | N/A |
| pMSCV-Blasticidin | [7] | Adgene plasmid #75085 |
| CSII-pEF-hDHB-mCherry | Laboratory of Dr Steven D. Cappell (Center for Cancer Research  National Cancer Institute,USA) | N/A |
| CSII-pEF-hGEMININ-mCherry | Laboratory of Dr Steven D. Cappell (Center for Cancer Research  National Cancer Institute,USA) | N/A |
| Software and algorithms | | |
| ImageJ | [8] | https://imagej.nih.gov/ij/ |
| Samtools | [9] | http://samtools.sourceforge.net/ |
| HTSeq | [10] | https://htseq.readthedocs.io/en/master/ |
| Deseq2 | [11] | https://bioconductor.org/packages/release/bioc/html/DESeq2.html |
| NIS-Elements | Nikon |  |
| SoftWoRx | Delta Vision |  |
| R studio |  | https://posit.co/ |
| MaxQuant software platform | [12] |  |
| Andromeda search engine | [13] |  |

1. Elbashir SM, Harborth J, Lendeckel W, Yalcin A, Weber K, Tuschl T: **Duplexes of 21-nucleotide RNAs mediate RNA interference in cultured mammalian cells.** *Nature* 2001, **411:**494-498.

2. Booth DG, Takagi M, Sanchez-Pulido L, Petfalski E, Vargiu G, Samejima K, Imamoto N, Ponting CP, Tollervey D, Earnshaw WC, Vagnarelli P: **Ki-67 is a PP1-interacting protein that organises the mitotic chromosome periphery.** *Elife* 2014, **3:**e01641.

3. Cuylen S, Blaukopf C, Politi AZ, Muller-Reichert T, Neumann B, Poser I, Ellenberg J, Hyman AA, Gerlich DW: **Ki-67 acts as a biological surfactant to disperse mitotic chromosomes.** *Nature* 2016, **535:**308-312.

4. Ran FA, Hsu PD, Wright J, Agarwala V, Scott DA, Zhang F: **Genome engineering using the CRISPR-Cas9 system.** *Nat Protoc* 2013, **8:**2281-2308.

5. Natsume T, Kiyomitsu T, Saga Y, Kanemaki MT: **Rapid Protein Depletion in Human Cells by Auxin-Inducible Degron Tagging with Short Homology Donors.** *Cell Rep* 2016, **15:**210-218.

6. Ariotti N, Rae J, Giles N, Martel N, Sierecki E, Gambin Y, Hall TE, Parton RG: **Ultrastructural localisation of protein interactions using conditionally stable nanobodies.** *PLoS Biol* 2018, **16:**e2005473.

7. Kendall J, Liu Q, Bakleh A, Krasnitz A, Nguyen KC, Lakshmi B, Gerald WL, Powers S, Mu D: **Oncogenic cooperation and coamplification of developmental transcription factor genes in lung cancer.** *Proc Natl Acad Sci U S A* 2007, **104:**16663-16668.

8. Schneider CA, Rasband WS, Eliceiri KW: **NIH Image to ImageJ: 25 years of image analysis.** *Nat Methods* 2012, **9:**671-675.

9. Li H, Handsaker B, Wysoker A, Fennell T, Ruan J, Homer N, Marth G, Abecasis G, Durbin R, Genome Project Data Processing S: **The Sequence Alignment/Map format and SAMtools.** *Bioinformatics* 2009, **25:**2078-2079.

10. Anders S, Pyl PT, Huber W: **HTSeq--a Python framework to work with high-throughput sequencing data.** *Bioinformatics* 2015, **31:**166-169.

11. Love MI, Huber W, Anders S: **Moderated estimation of fold change and dispersion for RNA-seq data with DESeq2.** *Genome Biol* 2014, **15:**550.

12. Cox J, Mann M: **MaxQuant enables high peptide identification rates, individualized p.p.b.-range mass accuracies and proteome-wide protein quantification.** *Nat Biotechnol* 2008, **26:**1367-1372.

13. Cox J, Neuhauser N, Michalski A, Scheltema RA, Olsen JV, Mann M: **Andromeda: a peptide search engine integrated into the MaxQuant environment.** *J Proteome Res* 2011, **10:**1794-1805.
