# Supplementary material for: Ki-67 is necessary during DNA replication for fork protection and genome stability
Source: Genome Biol. 2024 Apr 22;25:105. doi: 10.1186/s13059-024-03243-5 (PMC11034166; doi:10.1186/s13059-024-03243-5)
Supplement: Supplementary file 3 — Additional file 3. Uncropped western blots for Figure 2A, C, E; Figure 4F; Figure 5F-H, J; Additional file 1 Fig s1C, P; s2E-I; s4B; s5F-G. [file 13059_2024_3243_MOESM3_ESM.docx]

**Uncropped Western Blots**

Figure 2

Figure 4, 5

Additional file Figure S1

Additional file Figure S4,5
