# Supplementary material for: Ki-67 is necessary during DNA replication for fork protection and genome stability
Source: Genome Biol. 2024 Apr 22;25:105. doi: 10.1186/s13059-024-03243-5 (PMC11034166; doi:10.1186/s13059-024-03243-5)
Supplement: Supplementary file 4 — Additional file 4. Review history. [file 13059_2024_3243_MOESM4_ESM.docx]

Review History

**First round of review**

**Reviewer 1**

**Were you able to assess all statistics in the manuscript, including the appropriateness of statistical tests used?**

No, I do not feel adequately qualified to assess the statistics.

**Comments to author:**

In this paper, the authors develop a new Ki-67 degron cell line and use it to show an apparent role for Ki-67 in progression into S-phase. Unfortunately, the paper is written in a biased manner that ignores much of the scientific literature, and the data and interpretations are sufficiently flawed as to preclude their publication. The problems are numerous: below is a non-exhaustive list of my major reservations:

1. The cell line that the authors generated, HCT-116 with Ki-67 tagged with an auxin inducible degron and mClover (although supplementary figure 1 shows GFP) and doxycycline-inducible OsTIR1 E3 ligase (which is not described in the methods), is not well characterised in terms of the cell cycle, and appears to behave abnormally. It produces results which are different from 1) the 2016 study of Takagi et al (inexplicably not cited) using an ostensibly identical endogenous Ki-67 AID/mClover construction, also in HCT-116 cells, which did not report any cell cycle defects upon induced Ki-67 degradation and in which Ki-67 degradation appeared far more efficient, and 2) the paper from the Van Steensel lab (Van Schaik et al, EMBO Rep, 2022), which did not reveal any defects in the G1/S transition or S-phase progression using the Takagi Ki-67 degron cell line or a similar RPE1 cell line, where Ki-67 degradation was efficiently induced in cells synchronised in S-phase, as in the current manuscript. On the other hand, the Kaufman lab also used the Takagi HCT-116 Ki-67 AID cell line and reported a strong DNA damage response (Garwain et al., PNAS, 2021, also strangely not cited), apparently resulting from defects in mitosis but not S-phase. In this paper, the new HCT-116 Ki-67 AID/mClover cell line generated presents apparent non-specific cell cycle defects: figure 1A shows that in the absence of auxin this cell line proliferates less well than the control line, while addition of doxycycline alone, which should not have any effect in the absence of auxin, apparently reduces cell proliferation as Ki-67 expression is halved (supplementary figure S1C). Furthermore, the effects of auxin are not convincing: compared to doxycycline alone, auxin only elicits a slight further reduction in Ki-67 in the population (supplementary figure 1C). And in figure 5C, the nucleus that is out of the centre of the field (Ki-67-AID +Auxin, left bottom corner) is positive for Ki-67. In other words, the induced Ki-67 degradation does not work well comlpared to earlier apparently identical constructions. Yet the authors justify the necessity to re-make the same cell line by claiming that the cell line generated by Takagi et al. and employed in their 2018 paper showed that "some cells are not responding" (page 5, lines 10-12). This appears at best disingenuous. The Takagi 2018 paper (cited) used a clone that escaped the degradation as a control, but in their original paper (not cited), all Ki-67 expression was lost within three hours in the population they studied. If the authors want to claim that their results are reliable while those of Takagi et al., Garwain et al. and Van Schaik et al. are not, then they have to show very convincing evidence for this, which is not the case. In contrast, several labs have made a number of Ki-67 knockout cell lines (at least seven at the last count) that do not present any cell cycle defects, while the Depmap compendium of CRISPR screens shows an absence of effect on cell proliferation of complete ablation of Ki-67 in 1042 out of 1078 cell lines. While the authors attempt to claim that CRISPR knockouts might select for compensation mechanisms, this is not the case in CRISPR screens, in which common essential genes are easily identified. It is also not the case in siRNA or shRNA-induced knockdowns, which to date have not shown any effects of Ki-67 knockdown on cell cycle progression in any cell line studied, with the exception of the Sun et al. 2017 paper from the Kaufman lab, which was the first to claim a cell cycle role for Ki-67. In summary, the entire justification for this work is spurious, the cell line generated appears to be defective, and does not reproduce results from the scientific literature, which are not accurately reported in this paper. In my view, these problems, alone, are sufficient to question the reliability of this study.

2. If Ki-67 degradation genuinely slows the G1-S phase progression, then this should be visible without cell synchronisation, but experiments to test this have not been reported.

3. There are major flaws in interpretation that question the validity of the following conclusions. Firstly, the authors claim that Ki-67 triggers DNA damage, but one that specifically acts without phosphorylation of histone H2A.X (gamma-H2A.X). This claim is difficult to reconcile with the blot shown in figure 2A, where it is clear that the thymidine block itself triggers DNA damage, as seen by positive gamma-H2A.X signal, while degradation of Ki-67 evidently (and surprisingly) decreases this signal. Second, on page 8 is reported the discovery of interferon activation and DNA damage, while gamma H2A.X was not detected, yet the latter is the earliest and most ubiquitous mark of DNA damage and replication stress. The authors explain these results by invoking a gamma-H2A.X-independent pathway of DNA damage, which is not known to exist, and it would seem more logical to conclude that there is no DNA damage. Indeed, they then go on to say (Page 9) 'Lack of Ki-67 leads to … persistance of DNA damage' while gammaH2A.X is not present, and no other assessments of the DNA damage response (Rad51, 53BP1, phosphorylated CDK1, ATM/ATR, etc) or direct detection of DNA breaks (e.g. TUNEL assay) are presented. Next, the assertion that phosphorylated H2A.X is evicted from chromatin after attenuation of the DNA damage signaling is incorrect. It is dephosphorylated by phosphatases PP1, PP2A, PP4, depending on the source of damage (Moon et al, JBC 2010; Chowdhury et al, Mol Cell 2005; Chowdhury et al, Mol Cell 2008; Nakada et al, EMBO Rep 2008) and Pph3 in yeast (Keogh et al, Nature 2006). The slight decrease in gamma H2A.X in the +Auxin sample is not consistent with the claimed complete absence (still, compare with figure 2A), and the rescue with MG132 appears far more marginal than presented in the graph. Furthermore, the interpretations of results and references to the literature on page 13, lanes 14-21, do not make sense: "… indicate activation of MDC1 and TP53BP1 in response to DNA damage. Interestingly, this pathway seems to function either in regions where H2A.X is not present or in conditions where gH2A.X pathway is not working; our data of undetectable gH2A.X foci upon Ki-67 degradation at the G1/S boundary (Figure 2A and Figure 5C, D) are in agreement with the latter." This defies a large body of literature. 53BP1 colocalises with gamma H2A.X foci upon DNA damage (see e.g. Karanam et al, Mol Cell 2012; Fradet-Turcotte et al, Nature 2013). 53BP1 nuclear bodies contain gamma H2A.X, which is one of the first factors that initiates the recruitment of other factors that lead to the loading of 53BP1, and the ensuing cascade involves MDC1, RNF8, RNF168, the latter of which drives ubiquitination of K13-15 of H2A.X and gamma H2A.X that provide binding sites for 53BP1 (Mattiroli et al, Cell 2012; Fradet-Turcotte et al, Nature 2013). In any case, the authors did not perform any staining of 53BP1 in their cells to verify foci formation and their localisation. Worse still, the explanation given for the lack of gamma H2A.X signal, which is a ubiquitous marker of DNA damage and usually strongly correlates with positive comet assays, of an increase in HUWE-1 activity, is not demonstrated anywhere in any of the results. Finally, and perhaps most importantly, previously published work is ignored. This is especially true in the view of the Kaufman lab results (PNAS 2021), where, using the same cell line, HCT116, with the same degron system, strong gamma H2A.X induction was observed upon degradation of Ki-67; it is possible that this is also wrong, but it cannot be ignored.

4. Another conclusion that has been far too rapidly drawn in the present study is that no changes in chromatin organisation take place upon acute Ki-67 removal. This conclusion is solely based on a western blot assessing total levels of histone marks (Fig. 2E). The major problem with the conclusion, which underlies the interpretation of the whole paper, is that only total levels of histone marks were analysed but not their distribution or localisation. It was clearly demonstrated in previous publications that the total levels of histone modifications do not change upon loss of Ki-67, it is rather the organisation of chromatin that is altered. Without testing the chromatin compaction and organisation upon Ki-67 removal, no further conclusions on the direct role of Ki-67 in DNA replication can be drawn. All might be due to disorganisation of chromatin, which can take place within hours (as shown by van Schaik et al EMBO Rep 2022). Importantly, van Schaik et al. showed that Ki-67 competes with lamin B1 to bind centromeric chromatin regions. When Ki-67 is depleted, lamin B1 spreads to regions previously occupied by Ki-67. These are late-replicating heterochromatic regions. Genome-wide replication timing assays showed that depleting Ki-67 specifically delayed replication of these heterochromatic domains (and no others), coinciding with lamin occupation. This also suggests that, upon Ki-67 depletion, changes in chromatin organisation are rapid, and could easily be responsible for the phenotypes observed in the current paper.

5. In figure 2C Doxycycline appears to have a very similar effect on replication factor chromatin loading as Doxy+Auxin. It presumably itself affects cell proliferation, as seen with low PCNA loading, although why this should be the case is unclear. Therefore, one cannot talk about Ki-67 degradation-induced 'unloading' of ORC because its low presence likely reflects the low cycling of cells. Importantly, the similar cell line previously used by the Takagi, Van Steensel and Kaufman labs did not require doxycycline induction of OsTIR1, which was inserted into the AAVS locus under the CMV promoter. In this western blot it also does not seem that histone H3 is a reliable loading control, since there is less ORC, MCM3 and PCNA in the nuclei than in the whole cell lysate, yet there appears to be as much if not more histone H3. This does not make any sense as they are all exclusively nuclear. The only plausible interpretation is that the loading of samples which were blotted for H3 is not the same is that for the other proteins, and the pattern of lanes certainly suggests that these are not the same gels and thus an unacceptable loading control. This severely undermines my confidence in the reliability of these data.

6. That this cell line is behaving abnormally is also suggested by the RNA-seq data of Suppl. figure 2C -papers of both the Kaufman lab (PNAS 2021) and van Steensel lab (EMBO Rep) performed RNA-seq after induction of acute Ki-67 degradation, and they only found a handful of deregulated genes, whereas here there are hundreds. In particular, the van Steensel lab also used cell synchronisation in S-phase before inducing Ki-67 degradation, and they found only 3 genes whose expression was significantly deregulated.

7. It is impossible to figure out from the figure 1 and its legend what the difference is between the experiment presented in E-H and I-K. Judging by what is written in the text, there is an error in the scheme: in I-K, the treatment with Auxin is indicated 4h before release from thymidine while in the text the degradation of Ki67 is supposed to take place after the release. Thus, the scheme is incorrect.

8. In figure 2A- in the thymidine sample, Ki-67 is not well degraded; Palbociclib, in contrast, blocks Ki67 expression and is probably responsible for the majority of the decrease of the latter (and the tubulin control also appears reduced with auxin), and there is certainly no less p21 with palbociclib.

9. Page 10, Fig 4D - the major interactors of Ki-67, that reside in the main structure where Ki-67 is found in interphase (the nucleoli) are not mentioned. There is also no mention that mass spectrometry analysis of Ki-67 interactors was already performed (Schmidt et al, J Pathology 2003; Sobecki et al eLife 2016), and that MCM helicase components (MCM2, MCM3, MCM5, MCM6) as well as Treslin (TICRR) were previously identified.

10. It appears that loading is uneven in the blot shown in figure 4F, since asynchronous control cells apparently have much lower levels of MCM3 than the asynchronous APEX cells. This undermines the conclusion that Ki-67 interacts more with MCM3 upon thymidine block than in cycling cells.

11. The PLA assay shown in figure 4G, H looks somewhat non-specific as there are some dots in the cytoplasm, while the overlay of the PLA red signal and the green of GFP is not convincing. The interaction shown in figure 4K is probably over-interpreted since Ki-67 is present in late-replicating regions because these are heterochromatic regions, which are replicated at the end of S-phase.

12. The commentary on figure 6 is surprising: nucleolar proteins are found to be hyper-phosphorylated in the absence of Ki-67, but the authors conclude that "this should not come to a surprise since Ki-67 itself is a nucleolar protein". What do the authors mean?

13. One cannot conclude anything about a kinase activity simply by analysing by mass spectrometry the phosphorylation status of phospho-sites. The absence of a phospho-site in a mass spec analysis does not preclude its presence as detection problems might account for it.

14. A simple finding of several proteins implicated in chromosome cohesion in a phosphoproteomic screen does not justify such an affirmative conclusion of this section of the paper: "These analyses provide us with a picture whereby Ki-67 degradation … alters cohesion regulation" without any experiments performed to verify whether indeed any changes in cohesion can result from some phosphosites being altered.

15. Figure 7 shows some interesting findings but these are far too preliminary to conclude anything about replication or cohesion; it is likely that they also reflect differences in chromatin compaction. Furthermore, the nuclei chosen do not well illustrate the conclusions drawn: for example, in 7J the FISH area is shown to be much bigger with auxin, but the photo in 7K shows that it is smaller. Moreover, the relative intensities should be interpreted much more cautiously: in the photos shown in 7S, the signals are brighter with Auxin, yet there is no evidence that these are over-replicated.

16. The discussion is full of inaccuracies and unsupported statements. Here is a selection:

Page 17, line 4: what do the authors mean by Ki-67 being an 'election marker'?

The knowledge of the literature on Ki-67 seems to be very superficial. An example: (lines 13-17) they mention that xenografted Ki-67 KO cells cannot metastasise, citing a review by the Earnshaw lab, which is followed by stating that Ki-67 KO mice do not develop tumours - here, they cite the original paper (Mrouj et al 2021), disregarding that the previously invoked inability of Ki-67 KO xenografts to metastasise comes from the same paper.

Lines 33-37: The authors claim that their discovery that cells with acutely degraded cells progress more slowly into and through S-phase is new. However, this has already been claimed by the Kaufman lab.

Page 19, lanes 30/31: hyperphosphorylation of MDC1 and TP53BP1 is evoked: how can one talk about hyperphosphorylation based on phosphoproteomics and without a single blot?

Lane 40: "Ki-67 degradation causes the collapse of the replication forks", yet nowhere is this demonstrated.

Lanes 44-50: the finding of phosphorylated components of the cohesion complex leads to a conclusion that 'cohesin is possibly removed from the chromatin', but this was never tested.

Page 20, lines 4-6: "These data could also explain the link between Ki-67 levels and sensitivity to CDK inhibitors treatment" this appears to misunderstand that Ki-67 levels are a good readout of cell proliferation and thus indication for a likely response to CDK4/6 inhibitors (that in fact depends not on Ki-67 expression, which is present in all proliferating cell lines, but on RB, a target of CDKs).

Page 20, lines 9-11: "Ki-67 has been shown to bind PP1 but not many substrates have been identified apart from Ki-67 itself": is this supposed to mean that PP1 has no other substrates than Ki-67?

**Reviewer 2**

**Were you able to assess all statistics in the manuscript, including the appropriateness of statistical tests used?**

No, I do not feel adequately qualified to assess the statistics.

**Comments to author:**

The authors have explored the cell cycle contributions of Ki-67. Previous studies from this PI and others have shown that Ki-67 has prominent roles during mitosis: it is essential for formation of the perichromosomal layer, and thereby regulates mitotic chromosome structure and partitioning of nucleolar contents to daughter cells. A major question in the study of Ki-67 has been why phenotypes resulting from its loss appear to be so highly variable in different cell types. This study suggests that cells rapidly adapt to loss of Ki-67. This work also demonstrates an important new S phase function for this protein.

Many previous Ki-67 studies have used either RNAi-based mRNA depletion or degron-mediated protein depletion. Usually degron-mediated depletion experiments avoid adaptation problems, but Ki-67 appears to be an exceptional case. Earlier experiments with degron-tagged Ki-67 in a HCT116 colorectal cancer cell line derivative have demonstrated that Ki-67 shapes mitotic chromosome structure (pmid: 27610954), protects from mitotic DNA damage (pmid: 34353903) and regulates replication timing of pericentromeric heterochromatin (pmid: 36245428). However, the previous Ki-67-degron cell line used in those studies included a constitutively expressed TIR1 E3 ligase, and apparently the constant expression of TIR1 produced selective pressure for adaptation. Here, the authors have generated a new HCT116/Ki-67 degron line, placing TIR1 under inducible control of a TetR repressor. In these new cells, simultaneous treatment with doxycycline (to induce TIR1) and auxin (to trigger Ki-67 degradation) results in phenotypes not previously observed. Most notable are defects in S phase progression and DNA damage during that period.

This appears to be an important breakthrough in the study of Ki-67 and greatly extends the earlier observation of defects in pericentromeric replication upon Ki-67 depletion. Therefore, I am enthusiastic about these studies. However, I think there are several aspects of this work that warrant closer examination prior to publication, to clarify the extent of differences between the previous and the new degron cells:

The authors hint at rapid adaptation to the loss of Ki-67, observing that "the proliferation of cells lacking Ki-67 is severely impaired up to 96 h (Figure 1C); however, we noticed an increase in cell number at 120 h, suggesting that cells might adapt to the lack of Ki-67 over time." This is intriguing, but the hypothesis about adaptation should be tested directly by extending the time course in Fig. 1C. Does a subset of the cells indeed begin to proliferative more rapidly after long time periods in Doxycycline + Auxin? Can these late proliferating cells be isolated and analyzed? If so, what happens if the Dox + IAA are removed, and then reintroduced, are the S phase phenotypes lost?

Conversely, since the previous degron line did display reduced pericentromeric heterochromatin replication upon Ki-67 depletion, how different is the new cell line compared to the old regarding this phenotype? Also, in the old cell line, are the observed effects on the DNA replication proteins (e.g. reduced chromatin association (Fig. 2C-D) or PLA interaction with Mcm3 (Fig. 4G)) not observed?

In Fig. 1D, the time point examined is not mentioned. Indeed, it would be of interest to know how soon the S phase population disappears after auxin treatment.

I believe there is an error in how the diagram for Fig. 1I is drawn. In contrast to Fig. 1E, shouldn't the word "auxin" be placed at time = 0 here?

The text says that the "short MG132 treatment does not restore Ki-67 levels in cells" (p.11; Fig. 5C), but the drug concentrations and durations used are not mentioned.

A prediction of the HUWE1 data is that treatment with the HUWE1 inhibitor BI8626 will also increase the number of H2AX foci after acute Ki-67 depletion. Is that the case?

Fig. 7. At the time point analyzed, levels of BrdU incorporation in the RO-3306 blocked cells is not significantly reduced after Ki-67 depletion (Fig. 7A-C). This would suggest genome duplication is largely complete. So, do the reduced DNA-FISH signals result from reduced DNA synthesis (i.e. lower copy number) or instead reduced efficiencies of hybridization or detection in this assay? To distinguish these possibilities, quantitative measures of copy number of the loci analyzed should be done. For example, a quantitative PCR or ddPCR analysis could be done.

The comparison of Ki-67 expression levels with other genes is complicated by the fact that Ki-67 expression is highly correlated with cell proliferation. Therefore, the significance of the comparisons with the IFIT1 and STAT2 expression levels should be tested by asking whether other proliferation-associated gene expression signatures display similar correlations.

Places for text proofreading:

p.3, line 57: the phrase "and positioning" seems misplaced, I think it was meant to follow the words "compromised heterochromatin maintenance".

p.10, line 8: "not relying on antibodies of solubilisation problems". I think "of" should be "or" here.

Paul Kaufman

University of Massachusetts Chan Medical School

**Authors Response**

**Point-by-point responses to the reviewers’ comments:**

*Reply: Before proceeding to the point-to point response to the referees, we need to clarify about the cell line. As requested, we obtained the previously reported cell line from Dr Takagi who kindly shared it with us subjected to MTA.*

*We did several characterisations* ***that were missing in the literature****. One major aspect we discovered that is quite important in this context and can explain some differences between the cell lines, is the fact that Takagi’s cell line expresses a significant lower level of Ki-67 compared to both our and the parental HCT116^CMV:OSTR1^ that was used to generate the cell line. We have also compared the level of Ki-67 in the two parental cell lines and again verified that, while our cell line (HCT116-Ki-67-AID^TET-ON:OSTR1^) shows an homogeneous and comparable staining intensity as the parental cell line HCT116-Ki-67^TET-ON:OSTR1^ from which it was derived, the HCT116-Ki-67-AID^CMV:OSTR1^ has a reduced and uneven levels of Ki-67 compared to the parental HCT116-Ki-67^CMV:OSTR1^ (see Figure 1 A below).*

**Figure 1|** Comparison between our (KS) and Takagi’s (TK) cell lines.

A) Distribution of intensity levels of nuclear mClover in the two cell lines (left) in absence (Control) and in presence of the proteasome inhibitor MG132 (MG132); representative images of Ki-67 levels (right panels).

B) Rapresentatove images of Ki-67 immunofluorescence staining of the cell lines HCT116^TET-ON:OSTR1^, its derivative KS, the HCT116^CMV:OSTR1^ and its derivative TK. The images clearly show that the derivative TK expresses an uneven and reduced level of Ki-67 compared to the mother cell lines. To be noted also that in this cell line some nucleolar morphology is characteristic of a hioglky reduced Ki-67 level as we have previously reported (Booth et al, 2014 doi: 10.7554/elife.01641).

*The reason for this difference is the fact that Takagi’s cell line is constructed in the HCT116 expressing OSTR1 under the constitutive CMV promoter. This causes already degradation of the protein because the system is notoriously leaky (we did not obtain clones in that cell line however we only tried a few transfections; maybe you can get a clone that could survive). In fact, if the Takagi cell line is treated with the proteasome inhibitor MG132 for 4 h, the Ki-67 levels increase to a level comparable to the one in our cell line (Figure 1 B).*

*The leakiness of the system has been well known and reported by the group who developed the system (DOI: 10.1002/cpz1.219) as they state in their paper: “We recently established an improved system, called AID2, which involves expressing a mutant E3 ligase subunit, OSTIR1(F74G), and fusing a protein of interest to the mini-AID (mAID) tag, and that employs a new and more potent ligand, 5-phenyl-indole-3-acetic acid (5-Ph-IAA). The AID2 system overcomes some of the drawbacks associated with the original AID system, i.e., leaky degradation without auxin and the requirement of high auxin doses “.*

*On the contrary, the line we generated is based on the HCT116 that expresses OSTR1 under a DOX-regulated promoter; addition of DOX will induce OSTR1 expression: in fact, just addition of DOX causes a decrease in Ki-67 exactly for the same reason (supplementary Fig. 1 C). Therefore, our cell line provides a system to obtain acute depletion of the protein and the difference in Ki-67 levels is quite dramatic; on the other hand, the Takagi’s cell line has already about 60% reduced amount of Ki-67, and possibly adapted to grow at different basal levels; nevertheless, the major findings we have presented in this paper have also been reproduced with this cell line (delay in replication, interaction between MCM3 and Ki-67, and activation of the interferon response); we also think that this reduced Ki-67 expression could be the reason for seeing little differences in the RNA-seq experiments that were conducted using this cell line because the change in protein level is less dramatic than the one obtained with the inducible system we have used.*

*We have also noticed that this cell line presents a higher level of micronuclei compared to the original cell line and that has already an interferon response activated (as expected).*

*Having said this and clarified the differences, we think it still is a valid cell line for several investigations and It would be important resource in the future to understand cell adaptation to Ki-67 levels. We do not believe that any of the published data generated with this cell line are incorrect (maybe some caution should be taken about the RNA seq results).*

*We have not included these analyses in the revised manuscript as it is not our point to cast doubts on other people’s reagents. However, if you feel that it is important for the community to know, we could add it in the supplementary material.*

Reviewer #1:

In this paper, the authors develop a new Ki-67 degron cell line and use it to show an apparent role for Ki-67 in progression into S-phase. Unfortunately, the paper is written in a biased manner that ignores much of the scientific literature, and the data and interpretations are sufficiently flawed as to preclude their publication. The problems are numerous: below is a non-exhaustive list of my major reservations:

1 A) The cell line that the authors generated, HCT-116 with Ki-67 tagged with an auxin inducible degron and mClover (although supplementary figure 1 shows GFP) and doxycycline-inducible OsTIR1 E3 ligase (which is not described in the methods), is not well characterised in terms of the cell cycle and appears to behave abnormally.

*Reply: First, the referee has not carefully looked at the methods section where we clearly state in Methods- Experimental model and subject details: “The HCT116 OsTR1 stable expressing cell line was kindly provided by Dr Masato T. Kanemaki (University of Tokyo, Japan).” Page 22 lines 8-10. This information is also present alongside the reference of the publication in Supplementary Table 2 (Experimental Models: Cell line” section) Page 51 lines 36-40.*

*About the characterisation: This represents the best characterised cell line of Ki-67 AID that is available today. We have conducted 1) genotyping (Supplementary Figure 1 A, B), 2) Detailed growth curves with and without single drugs and combination of the drugs (Figure 1 C), 3) Flow Cytometry analyses (Figure 1 D), Immunofluorescence (Figure 1 B), and Western Blotting (Supplementary Figure 1 C). We are not sure what the reviewer means with “behave abnormally” as the cell line behaves very normally without the addition of DOX or Auxin.*

*The only other cell line available to the community with an AID tag has not been characterised at all (apart from the genotype). It was published by Takagi et al in 2016 (doi.org/10.1111/gtc.12420) and, as the Editor could easily verify, in this manuscript there are NO growth curves, NO Flow cytometry and Ki-67 DOES NOT degrade as well as in our cell line (Compare Figure 1 F of Takagi et al with Supplementary Figure 1 C of out Manuscript). ­­­The Takagi study did not provide any other characterisation of the cell line neither in this paper nor in the following papers.*

*In addition, we have obtained the cell line and done some characterisations that were missing in the literature. The outcome has been described at the beginning of this rebuttal letter.*

*Therefore, we can clearly state that our cell line is the most characterised one up to date.*

1 B) ...It produces results which are different from 1) the 2016 study of Takagi et al (inexplicably not cited) using an ostensibly identical endogenous Ki-67 AID/mClover construction, also in HCT-116 cells, which did not report any cell cycle defects upon induced Ki-67 degradation and in which Ki-67 degradation appeared far more efficient,

*Reply: The paper mentioned by the reviewer is focussed on mitotic chromosomes, this is not the topic of our study. Of course, if we look at mitotic chromosomes, we do see the same defects on chromosomes behaviour and structure that we have previously reported in 2014 (doi: 10.7554/elife.01641)(we were the first to publish these observations as Reviewer 2 had mentioned and acknowledged) and others have also reported afterwards.* ***The present study does not focus on mitosis.***

*However, just to clarify the point and satisfy the referee, we have:*

1) *repeated the experiment of G1/S block and release to monitor S phase entry with and without Auxin and we obtained exactly the same results we have reported for our cell line (Supplementary Figure 1 D);*
2) *shown below a panel of the mitotic phenotype observed in our cell line (this is not added to the manuscript because it would not be appropriate for the topic). As the referee can see, the clustering of chromosomes is indeed present in the cell line treated with auxin as previously reported (including our studies) and similarly, the cytoplasmic nucleolin aggregates are also present as we have previously reported.*

**Figure 2|** Representative images of mitotic cell from the HCT116^TET-ON OSTR1^-Ki-67-AID (the cell line generated in this study) in the absence (-) and presence (+) of auxin. The chromosome clustering phenotype is evident in presence of auxin, when Ki-67 is degraded. Moreover, the localisation of nucleolin in these cells is impaired as we previously described (Booth et al, 2014 doi: 10.7554/elife.01641).

*Moreover, in the paper indicated by the referee there is no report of cell cycle defects* ***because they have not conducted any cell cycle analyses with this cell line****, as I mentioned in my previous comments. In this respect, the reviewer tries to make a conclusion on a paper they have not either carefully read or remember.*

*About the Citation: We have referred to this paper in the introduction” by quoting another paper by the same author using the same cell line. I believe it counts as acknowledgment. The text reads as follows: “More recently, the use of a degron system has allowed to dissect more specifically some of the functions of Ki-67 in mitosis [10], but the cell line available to the community, although extremely valuable for single cell studies, appears to have a varied response to auxin where some cells are not responding [10], making any biochemical approach difficult to interpret. (Page 5 lines 5-12).*

*In this paper, the authors used the same cell line but also clearly showed that the cell line does not degrade efficiently as the authors themselves comment “In control cells in which both Ki-67 and SMC2 escaped from degradation during the imaging period, mitotic chromosomes kept their structural integrity (Fig. 4C) https://doi.org/10.1242/jcs.212092.”*

*Just to reiterate, in this study the authors* ***only analysed mitosis and there is no other cell cycle characterisation.***

*Please refer to the comment before: Ki-67 does not degrade as well as in our cell line (Compare Figure 1 F of Takagi et al 2016 with Supplementary Figure 1 C of out Manuscript).*

1 C) …. the paper from the Van Steensel lab (Van Schaik et al, EMBO Rep, 2022), which did not reveal any defects in the G1/S transition or S-phase progression using the Takagi Ki-67 degron cell line or a similar RPE1 cell line, where Ki-67 degradation was efficiently induced in cells synchronised in S-phase, as in the current manuscript.

*Reply: As the editor could verify, Van Steensel lab study* ***did find a difference*** *(but they did not use the same assay nor a synchronisation protocol); as the Authors state in the paper: “Next, we asked whether Ki-67 depletion affected the replication timing of the regions that it interacts with. We tested this by Repli-seq (Marchal et al, 2018), 24 h after Ki-67 depletion in (unsynchronized) HCT116 cells (Fig 5D and E). While replication timing across most of the genome remained unaffected by this depletion,* ***we did observe a small but consistent delay in replication timing in peri-centromeric regions (Fig 5F and G). Centromeric sequences, which cannot be uniquely mapped to individual chromosomes, also showed a delayed replication timing upon Ki-67 depletion (Fig EV4C)****. The replication timing observations are different from Lamin B1 interactions that increased near centromeres but also on other domains with high Ki-67 scores (Fig 5D, purple arrows, Fig EV4D and E). Thus, besides its known roles in mitosis,* ***Ki-67 specifically affects the replication timing of (peri-)centromeric regions in interphase.***

*In this respect, I think we and the Van Steensel group do agree and the studies are not at odd. Our phenotype is stronger because 1) they used the cell line previously made where the degradation is not as efficient as ours 2) we used a synchronisation protocol instead of an asynchronous population.*

*Moreover, our results were also confirmed using 2 previously validated SiRNA oligos (as we show in Supplementary Figure 1 F) and we reproduced the same results also using the Takagi’s cell line (Supplementary Figure 1 D).*

1 D) On the other hand, the Kaufman lab also used the Takagi HCT-116 Ki-67 AID cell line and reported a strong DNA damage response (Garwain et al., PNAS, 2021, also strangely not cited), apparently resulting from defects in mitosis but not S-phase.

*Reply:* ***Again, this comment is inaccurate****. If the Editor looks at the paper quoted by the reviewer, you can clearly see that Ki-67 IAA cells do not have any gH2AX foci in S phase (Figure 2 Panel C). The foci are present only in mitosis. These data agree perfectly with what we report: our paper is focused on S phase and not on mitosis.*

1 E) this cell line proliferates less well than the control line, while addition of doxycycline alone which should not have any effect in the absence of auxin, apparently reduces cell proliferation as Ki-67 expression is halved (supplementary figure S1C). Furthermore, the effects of auxin are not convincing: compared to doxycycline alone, auxin only elicits a slight further reduction in Ki-67 in the population (supplementary figure 1C).

*Reply:* ***This statement is incorrect****: This is an inducible system, and the addition of Dox will induce OSTR1. This system is known for having a leaky degradation without auxin in the presence of OSTR1. This is also the reason that the Takagi’s cell line expresses much less Ki-67 as OSTR1 is expressed using a constitutive promoter (See Figure 1 at the beginning of the rebuttal letter).*

*In fact, for this reason, the Kanemaki group has developed a new system called AID2. This system was not available when we started the project. The following paragraph is from the paper by Kanemaki (DOI: 10.1002/cpz1.219).*

*“We recently established an improved system, called AID2, which involves expressing a mutant E3 ligase subunit, OsTIR1(F74G), and fusing a protein of interest to the mini-AID (mAID) tag, and that employs a new and more potent ligand, 5-phenyl-indole-3-acetic acid (5-Ph-IAA). The AID2 system overcomes some of the drawbacks associated with the original AID system, i.e., leaky degradation without auxin and the requirement of high auxin doses “.*

***Clearly the Referee does not have knowledge of the technology in question****.*

1 F) If the authors want to claim that their results are reliable while those of Takagi et al., Garwain et al. and Van Schaik et al. are not...

*Reply: This is not what we claim anywhere in the manuscript. We are not sure why the referee is so angry and wants to make the editor believe that we discredit other people’s work.* ***We are not claiming that the results in those papers are not reliable.*** *We have focussed on a completely different type of analyses that were not conducted by any of the authors the referee refers to. We found a mechanism that other papers did not investigate.* ***We do not describe mitotic phenotypes.***

1 G) In summary, the entire justification for this work is spurious, the cell line generated appears to be defective, and does not reproduce results from the scientific literature, which are not accurately reported in this paper. In my view, these problems, alone, are sufficient to question the reliability of this study.

*Reply: I hope the Editor agrees that, considering all the evidence we provide that clearly contradict all the statements made by the referee****, this conclusion is unfounded. Every single statement in the previous sentence is incorrect.***

***Here we also provide evidence that the only Ki-67-degron cell line available to the community is indeed a cell line already adapted to low level of Ki-67 (only 40%) of the amount the cell line should express (Figure 1 of the rebuttal letter). In this respect, little differences observed in previous studies using this cell line should be carefully interpreted.***

***There is also a general concern about the scientific approach here. Why is considered so negative (in the reviewer’s opinion) to generate different cell lines with similar tools? This should give more reliability to discoveries and provide more tools to the community. We have generated several cell lines APEX2-AID, APEX2, V5-AID and ALL behave in the same way.***

***We have now provided the community with a cell line that expresses the correct amount of Ki-67 and has not been adapted to low levels.***

***Finally, I want to draw the attention on the language: the adjective “spurious” in the English dictionary is a synonym of counterfeit: this is a big allegation to make to the work of colleagues, especially when the reviewer has clearly shown to have looked at the paper in a superficial manner and not to know the content of papers that have been quoted inaccurately. I would suggest that the referee would refrain in the future from using such choice of words and carefully check their meaning. This is totally unprofessional.***

2. If Ki-67 degradation genuinely slows the G1-S phase progression, then this should be visible without cell synchronisation, but experiments to test this have not been reported.

*Reply: Here the referee did not look at the paper carefully. We have indeed reported this by showing the cell cycle profile by flow cytometry and shown in Figure 1 D. The corresponding text reads as follows “Cell cycle analyses by flow cytometry did not show sign of apoptosis but clearly revealed that the proportion of cells in S phase was greatly diminished (Figure 1 D).”*

*Moreover, we have shown the same phenotype using two independent and validated SiRNA oligos (Supplementary Figure 1 F – Previous Supplementary Figure 1 E). In the revised manuscript, we have also repeated the same experiment using the Takagi cell line and we obtained the same results (Supplementary Figure 1 D).*

3 A) Firstly, the authors claim that Ki-67 triggers DNA damage, but one that specifically acts without phosphorylation of histone H2A.X (gamma-H2A.X). This claim is difficult to reconcile with the blot shown in figure 2A, where it is clear that the thymidine block itself triggers DNA damage, as seen by positive gamma-H2A.X signal, while degradation of Ki-67 evidently (and surprisingly) decreases this signal.

*Reply: I am not sure what the referee is surprised by. The staining is the base line and clearly Ki-67 degradation causes a decrease in signal.*

*The referee should also look at the images and quantifications presented in Figure 5 C and D where gH2AX foci were counted. This is also in agreement with the observations by Kaufman lab (Garwain et al., PNAS, 2021) where they reported that cells with degraded Ki-67 do not have gH2AX foci in S phase (Garwain et al., PNAS, 2021- Figure 2 Panel C).*

*To also note that, in order to exclude that the damage was triggered by the Thymidine treatment, we have used the CDK4/6 inhibitor Palbociclib (that does not induce damage) and we could obtain the same results (Figure 2 A and Supplementary Figure 1 E).*

*We have added some extra text to link these findings. Now the text reads as follows:*

“The observation of lack of γH2AX foci in S phase for Ki-67 depleted cells agrees with previous work where γH2AX signals were observed only in the mitotic stage of auxin-treated cells but not in S phase [32].”

3 B) Second, on page 8 is reported the discovery of interferon activation and DNA damage, while gamma H2A.X was not detected, yet the latter is the earliest and most ubiquitous mark of DNA damage and replication stress. The authors explain these results by invoking a gamma-H2A.X-independent pathway of DNA damage, which is not known to exist, and it would seem more logical to conclude that there is no DNA damage.

*Reply: This could be the logical conclusion for the referee* ***if anyone decided to ignore the data*** ***we presented*** *in Figure 3 F and G where we show that indeed the damage exists as we used a direct assay to analyse the damage (comet assay). Therefore, the damage exists and we cannot ignore it.*

*About the comment on the non-existing pathway: This statement is incorrect. Work by the Jackson lab has clearly demonstrated this. An MDC1-mediated pathway that promotes association with DNA in an H2AX-independent manner. The Editor can confirm the correctness of our statement: Salguero, I., et al. MDC1 PST-repeat region promotes histone H2AX-independent chromatin association and DNA damage tolerance. Nat Commun 10, 5191 (2019).DOI/10.1038/s41467-019-12929-5).*

3 C) ...and no other assessments of the DNA damage response (Rad51, 53BP1, phosphorylated CDK1, ATM/ATR, etc) or direct detection of DNA breaks (e.g. TUNEL assay) are presented.

*Reply: Here the referee shows not to have read the paper carefully as this statement is again incorrect: we have a full phosphoproteomic that detects 53BP1 phosphorylations linked to DNA damage (Figure 6 D) and we have a COMET assay to detect damage (Figure 3 F and G).*

3 D) The slight decrease in gamma H2A.X in the +Auxin sample is not consistent with the claimed complete absence (still, compare with figure 2A), and the rescue with MG132 appears far more marginal than presented in the graph.

*Reply: Here the referee is confusing two major points: 1) the blots represent the entire cell extract and does not provide any evidence that gH2AX foci localised in the nucleus exist. 2) the band seen is EXACTLY at the same intensity in control cells even on Palbociclib (which does not cause DNA damage) therefore the band represents the basal level and does not produce any kind of foci information as shown by IF in Figure 5 C (and quantified in D). The changes with MG132 are major rather than marginal as can be appreciated. These experiments have been repeated several times independently, blindly scored and statistically analysed. The statistical analyses show a highly significant difference.*

3 E) …and TP53BP1 in response to DNA damage. Interestingly, this pathway seems to function either in regions where H2A.X is not present or in conditions where gH2A.X pathway is not working; our data of undetectable gH2A.X foci upon Ki-67 degradation at the G1/S boundary (Figure 2A and Figure 5C, D) are in agreement with the latter." This defies a large body of literature. 53BP1 colocalises with gamma H2A.X foci upon DNA damage (see e.g. Karanam et al, Mol Cell 2012; Fradet-Turcotte et al, Nature 2013). 53BP1 nuclear bodies contain gamma H2A.X, which is one of the first factors that initiates the recruitment of other factors that lead to the loading of 53BP1, and the ensuing cascade involves MDC1, RNF8, RNF168, the latter of which drives ubiquitination of K13-15 of H2A.X and gamma H2A.X that provide binding sites for 53BP1 (Mattiroli et al, Cell 2012; Fradet-Turcotte et al, Nature 2013). In any case, the authors did not perform any staining of 53BP1 in their cells to verify foci

formation and their localisation.

*Reply: I am not sure why the referee keeps ignoring the paper by Steve Jackson’s lab (who is a well-respected scientist in the field of DNA repair); maybe I suggest the reviewer should read his paper DOI/10.1038/s41467-019-12929-5. The literature quoted, although extremely important and pioneer in the field is from 2013 and the article Jackson wrote is more recent (2019). New discoveries happen all the time.*

3 F) Worse still, the explanation given for the lack of gamma H2A.X signal, which is a ubiquitous marker of DNA damage and usually strongly correlates with positive comet assays, of an increase in HUWE-1 activity, is not demonstrated anywhere in any of the results.

*Reply: Here the referee is incorrect again. We have an entire figure dedicated to assessing the activity of Huwe1 (Figure 5 E-I) and Supplementary Figure 4 A and B. We have tested all the known Huwe1 substrates (CHK1, H2AX, p53) important at this stage of the cell cycle and shown a reduction (for all) that can be ameliorated by either blocking the proteasome activity with MG132 or blocking the activity of the ligase using a specific inhibitor BI8626. These data demonstrate that all the known HUWE-1 substrates are reduced upon Ki-67degradation, that their decrease is due to the proteasome (MG132 treatment) and not transcription (no difference in mRNA levels), and finally that inhibition of HUWE-1 specifically increases their levels and the number of cells with gH2AX foci.*

3 G) This is especially true in the view of the Kaufman lab results (PNAS 2021), where, using the same cell line, HCT116, with the same degron system, strong gamma H2A.X induction was observed upon degradation of Ki-67; it is possible that this is also wrong, but it cannot be ignored.

*Reply: I have already commented on this. If the Editor could look at the paper quoted by the reviewer, they could clearly see* ***that Ki-67 IAA cells do not have any gH2AX foci in the S phase*** *(Figure 2 Panel C). The foci are present in mitosis. These data agree perfectly with what we report: our paper is focused on the S phase and not mitosis. We did not comment on it because we did not look at mitosis or G2. We are happy to mention it somewhere.*

4 A) Another conclusion that has been far too rapidly drawn in the present study is that no changes in chromatin organisation take place upon acute Ki-67 removal. This conclusion is solely based on a western blot assessing total levels of histone marks (Fig. 2E). The major problem with the conclusion, which underlies the interpretation of the whole paper, is that only total levels of histone marks were analysed but not their distribution or localisation. It was clearly demonstrated in previous publications that the total levels of histone modifications do not change upon loss of Ki-67, it is rather the organisation of chromatin that is altered. Without testing the chromatin compaction and organisation upon Ki-67 removal, no further conclusions on the direct role of Ki-67 in DNA replication can be drawn.

*Reply: First of all, this is a minor point and does not underlie the interpretation of the whole paper as claimed (if the referee has read the paper correctly). We have described the experiments we have conducted. The referee could have asked for additional; however, the paper the referee quoted (*[*https://doi.org/10.15252/embr.202255782*](https://doi.org/10.15252/embr.202255782)*) shows that Ki-67 does not detectably control 3D genome interactions, which normally is a proxy for chromatin organisation, in interphase (all Figure 4 dedicated to this).*

*So now do they want us to repeat the experiments that others have already done and then criticizing us for doing them? This seems to contradict the line played so far.*

*We can refer to this paper.*

5 A) In figure 2C Doxycycline appears to have a very similar effect on replication factor chromatin loading as Doxy+Auxin. It presumably itself affects cell proliferation, as seen with low PCNA loading, although why this should be the case is unclear. Therefore, one cannot talk about Ki-67 degradation-induced 'unloading' of ORC because its low presence likely reflects the low cycling of cells.

*Reply: Here we have already explained about the leakiness of the degradation once OSTR1 is present (see reply to Point 1 E).*

*In addition, this is not a consequence of cell proliferation as we have conducted all these experiments at the G1/S boundary and when cells have already transited through the restriction point. As such, the referee should compare the transcription level of the all the MCMs (that do not change) and the protein level in total extracts (that do not change).*

5 B) The only plausible interpretation is that the loading of samples which were blotted for H3

is not the same is that for the other proteins, and the pattern of lanes certainly suggests that these are not the same gels and thus an unacceptable loading control. This severely undermines my confidence in the reliability of these data.

*Reply: The H3 is used as loading control for each membrane that is processed, and it is used to normalise the other markers* ***within the same membrane****. All our blots are processed with fluorescent antibodies, captured in the linear dynamic range and quantified/statistical analysed. You cannot be more accurate than this.*

6. That this cell line is behaving abnormally is also suggested by the RNA-seq data of Suppl. figure 2C -papers of both the Kaufman lab (PNAS 2021) and van Steensel lab (EMBO Rep) performed RNA-seq after induction of acute Ki-67 degradation, and they only found a handful of deregulated genes, whereas here there are hundreds. In particular, the van Steensel lab also used cell synchronisation in S-phase before inducing Ki-67 degradation, and they found only 3 genes whose expression was significantly deregulated.

*Reply: Again, this point does not make sense. First, you cannot compare the transcriptomics results conducted at a specific stage of the cell cycle with the ones conducted with an asynchronous population. The results can be and ARE quite different: we have clearly shown this by comparing the differences between the degradation in mitosis (supplementary Figure 2 A-C) versus the degradation in G1/S (Figure 3 A, B, supplementary Figure 2D).*

*We actually do agree with van Steensel lab that there are not many transcriptional changes at the G1/S although we see more differentially expressed genes than they reported. This could be due to two factors 1) the threshold for selecting differentially expressed genes and 2) the extent of degradation. If the previous cell line had a level of heterogeneity in the degradation, the effect would be expected to be less prominent.*

*Having now also* ***characterised carefully for the first time*** *the Takagi’s cell line and shown that it has a reduced level of Ki-67 and activated interferon response, we can explain why the differences (including the interferon response we have identified) between with and without auxin are so small.*

*In addition, and ignored by the referee, it has to be noted that work in mice and in human cells (*[*https://doi.org/10.1073/pnas.2026507118*](https://doi.org/10.1073/pnas.2026507118)*) by Daniel Fisher’s lab, reported MANY transcriptional changes (Figure 3 A-C) and, among those, the interferon-gamma and alpha were among the highest reported (Figure 3 E and H).*

*Therefore, this seems perfectly aligned with our findings. We have identified the mechanism.*

*We have added more information within the text in the discussion about this point and the text reads as follows: “*Interestingly, the interferon-gamma and alpha were also found among the highest deregulated genes in 4T1 Mki67−/− and MDA-MB-231 MKI67−/− cells [19]. Therefore, the activation of interferon pathway appears to be a general and important feature of Ki-67 depletion.”

*If we degrade Ki-67 in mitosis and analyse the transcriptome in G1 we do see the same differences reported by Kaufman in doi: 10.1128/mcb.00569-16 (data presented in Supplementary Figure 2 B and commented in the discussion). Therefore, there is not the “strange behaviour” as the reviewer wants to make the Editor believe.*

9. Page 10, Fig 4D - the major interactors of Ki-67, that reside in the main structure where Ki-67 is found in interphase (the nucleoli) are not mentioned. There is also no mention that mass spectrometry analysis of Ki-67 interactors was already performed (Schmidt et al, J Pathology 2003; Sobecki et al eLife 2016), and that MCM helicase components (MCM2, MCM3, MCM5, MCM6) as well as Treslin (TICRR) were previously identified.

*Reply: Actually, we commented on this, and they are presented as GO terms in the related Figure. We can easily add another colour to indicate all the nucleolar proteins.*

*We can add the references, but those experiments were not conducted at a specific stage of the cell cycle, and they were obtained by overexpressing a FLAG-tagged version of Ki-67. Here we present a major dataset using a* ***proximity biotinylation approach of the endogenous protein that nobody has ever performed****. Our dataset has identified MCMs but not treslin suggesting that Teslin might not be in a close contact with Ki-67 at least in this stage of the cell cycle.*

*However, we have modified the text as follows:*

*“Interestingly, MCM components were previously identified in a study where a FLAG-tagged versions of full-length human Ki-67 was overexpressed in U2OS cells, and pulled downs from nuclear extracts with anti-FLAG antibody was carried out in an asynchronous population [13], however, these interactions were never confirmed by any other approach.”*

10. It appears that loading is uneven in the blot shown in figure 4F, since asynchronous control cells apparently have much lower levels of MCM3 than the asynchronous APEX cells. This undermines the conclusion that Ki-67 interacts more with MCM3 upon thymidine block than in cycling cells.

*Reply: 1) of course, cells in G1/S have more MCM than cycling cells 2) the pulldown had to be measured as enrichment over the input and clearly, this is the case. 3) the blot is just to verify the big dataset* ***we obtained by SILAC mass spec*** *(which is quantitative).*

11. The PLA assay shown in figure 4G, H looks somewhat non-specific as there are some dots in the cytoplasm, while the overlay of the PLA red signal and the green of GFP is not convincing. The interaction shown in figure 4K is probably over-interpreted since Ki-67 is present in late-replicating regions because these are heterochromatic regions, which are replicated at the end of S-phase.

*Reply: Clearly, the reviewer is not familiar with PLA assays. Like any antibody staining, there will be a certain level of background but 99.9% of the signals are nuclear (overlaps with DAPI).*

*Second, here we do not show the overlaps with Ki-67. Ki-67 is enriched at the nucleoli but, as the reviewer can see the interaction with the MCM occurs in the nuclear space. In fact, Ki-67 is indeed present (although at lower levels) in the nuclear space (Supplementary Figure 3C): this is where the interaction occurs.*

***This is exactly the point we are making****: in this case we do agree.*

12. The commentary on figure 6 is surprising: nucleolar proteins are found to be hyper-phosphorylated in the absence of Ki-67, but the authors conclude that "this should not come to a surprise since Ki-67 itself is a nucleolar protein". What do the authors mean?

*Reply: I do not understand the comment here. Ki-67 is a nucleolar protein, when you remove it is it a surprise that signalling will change in the compartment (unless it is a completely useless protein)? I would have been very surprised if none of the nucleolar proteins would have shown any difference. The nucleolus is a very active compartment.*

13. One cannot conclude anything about a kinase activity simply by analysing by mass spectrometry the phosphorylation status of phospho-sites. The absence of a phospho-site in a mass spec analysis does not preclude its presence as detection problems might account for it.

*Reply: Two points here:*

*1) I think here the reviewer has not paid attention to the experiment: it is a SILAC-based experiment and therefore we are measuring the ratio of the phospho peptides. If you find a ratio, it is because the peptide flies and it is detected in the sample. We cannot make and we are not making any comments on the peptides that are not detected.*

*2) So, if you detect changes in phosphorylation ratios when comparing two samples (these studies are made mixing cells differentially labelled 1:1– remember that is SILAC and normalised), it is because either this site is less phosphorylated or it is more de-phosphorylated. The samples are normalised for abundance.*

*In addition,* ***we have tested directly the kinase activity*** *using a cell line we made with a reporter for CDK2 activity and we have clearly shown that this kinase is indeed inactive (Supplementary Figure 4C-D).*

14. A simple finding of several proteins implicated in chromosome cohesion in a phosphoproteomic screen does not justify such an affirmative conclusion of this section of the paper: "These analyses provide us with a picture whereby Ki-67 degradation … alters cohesion regulation" without any experiments performed to verify whether indeed any changes in cohesion can result from some phosphosites being altered.

*Reply: First is not a simple finding; second, the phosphorylation of these proteins has been shown to alter cohesion biology* (we have all the related references in the paper); third we have measured the distance of the loci after replication (a classic test to measure sister chromatid cohesion) (Figure 7).

15. Figure 7 shows some interesting findings but these are far too preliminary to conclude anything about replication or cohesion; it is likely that they also reflect differences in chromatin compaction. Furthermore, the nuclei chosen do not well illustrate the conclusions drawn: for example, in 7J the FISH area is shown to be much bigger with auxin, but the photo in 7K shows that it is smaller. Moreover, the relative intensities should be interpreted much more cautiously: in the photos shown in 7S, the signals are brighter with Auxin, yet there is no evidence that these are over-replicated.

*Reply: We are really surprised that the reviewer finds something interesting.*

*We do disagree with the referee because the experiment* ***does prove that replication is incorrect****: the only way you can have less signal of the probes is that you have less copies. We have maintained the color scaling to represent the signal intensity. If you want an image representing the area, as the signal is spread, you need to increase the scaling for the Auxin: This is not a good practice.*

*For the single probe: it is the ratio between the intensity of the sister chromatids that matters. This quantification was conducted based on many cells from different experiments and shown as graphs.*

16. The discussion is full of inaccuracies and unsupported statements. Here is a selection:

Page 17, line 4: what do the authors mean by Ki-67 being an 'election marker'?

*Reply: We changed “election” with “standard”*

The knowledge of the literature on Ki-67 seems to be very superficial. An example: (lines 13-17) they mention that xenografted Ki-67 KO cells cannot metastasise, citing a review by the Earnshaw lab, which is followed by stating that Ki-67 KO mice do not develop tumours - here, they cite the original paper (Mrouj et al 2021), disregarding that the previously invoked inability of Ki-67 KO xenografts to metastasise comes from the same paper.

*Reply: I tend to disagree with the reviewer. We have contributed to the field with seminal discoveries (as acknowledge by referee 2) and we have written many reviews and editorials, therefore we know the literature on Ki-67. I am afraid the referee is somehow confused on the contents of some papers as shown in several of their comments. We think that the Earnshaw’s review did a very good job in summarising this point. Normally you do not quote twice the same reference in a sentence but, if requested, we have duplicated the Mrouj’s reference.*

Lines 33-37: The authors claim that their discovery that cells with acutely degraded cells progress more slowly into and through S-phase is new. However, this has already been claimed by the Kaufman lab.

*Reply: First of all, this statement is incorrect because:*

*1) we have mentioned this in the paper:* *“This observation agrees with data obtained in hTERT-RPE1 cells where depletion of Ki-67 by RNAi delayed S-phase entry upon arrest with hydroxyurea [27].”*

*2) However, it has to be noted that, because of the RNAi approached used in the mentioned publication and the effect caused in the exit from mitosis without Ki-67 (our data presented in Supplementary Figure 2 A, B), the experiments by Sun et al cannot be conclusive of a direct effect of Ki-67 on DNA replication. Of course, if you downregulate the replication machinery genes, cells do not initiate replication, or they do it badly,* ***but it is quite an important and different point.*** *We have also discussed this point.*

*In this respect, I think we can make the claim.*

Page 19, lanes 30/31: hyperphosphorylation of MDC1 and TP53BP1 is evoked: how can one talk about hyperphosphorylation based on phosphoproteomics and without a single blot?

*Reply: Differential phosphoproteomic is the experiment we did, and we reported the results we have found.*

Lane 40: "Ki-67 degradation causes the collapse of the replication forks", yet nowhere is this demonstrated.

*Reply: We show that several components of the replication machinery are lost from the chromatin (Figure 2 C-D).*

Lanes 44-50: the finding of phosphorylated components of the cohesion complex leads to a conclusion that 'cohesin is possibly removed from the chromatin', but this was never tested.

*Reply: We demonstrate there is lack of cohesion in G2 following the replication without Ki-67 (Figure 7 L-S). Moreover, all the phosphorylation we have identified have been shown to be involved in the removal of cohesion.*

Page 20, lines 4-6: "These data could also explain the link between Ki-67 levels and sensitivity to CDK inhibitors treatment" this appears to misunderstand that Ki-67 levels are a good readout of cell proliferation and thus indication for a likely response to CDK4/6 inhibitors (that in fact depends not on Ki-67 expression, which is present in all proliferating cell lines, but on RB, a target of CDKs).

*Reply: Here the referee has misunderstood and makes assumptions.*

*First: CDK4/6 inhibitors do indeed decrease the levels of Ki-67 (known before and also proved in the data we provided in Figure 2 A); 2) Ki-67 level differs in proliferating cell lines to the point that cells can proliferate with it, so the link is not as obvious. However, without it, there are some underlying problems that lead to hypersensitivity to CDK inhibitors (and we predict that CDK2 inhibitors would be very effective, but this is all another paper).*

Page 20, lines 9-11: "Ki-67 has been shown to bind PP1 but not many substrates have been identified apart from Ki-67 itself": is this supposed to mean that PP1 has no other substrates than Ki-67?

*Reply: This is not what the sentence means. Maybe the referee does not know the PP1 biology. PP1 works in a complex with other proteins called targeting subunits or RIPPOs. Therefore, there is not a single PP1 but many PP1 holocomplexes that have particular substate specificities.*

*We have shown that Ki-67/PP1 is one of those and, so far, the only specific substrate identified for this homocomplex is Ki-67 itself.*

*I do not know if there are other Ki-67/PP1 substrates, nobody has ever identified any other ones, I do not have a magic sphere and therefore and I cannot answer the question.*

Reviewer #2:

*Reply: We thank the referee for the time spent in assessing our manuscript and for the constructive comments provided. We are also very pleased that such an expert in this field as Prof Kaufman*

*has recognised that this work represents “an important breakthrough in the study of Ki-67 and greatly extends the earlier observation of defects in pericentromeric replication upon Ki-67 depletion.”*

*We have tried our best to incorporate the referee’s comments and I hope the referee would be satisfied of our responses.*

*Please find below our response to the comments raised (in blue) and how the manuscript has been modified (in green).*

1) The authors hint at rapid adaptation to the loss of Ki-67, observing that "the proliferation of cells lacking Ki-67 is severely impaired up to 96 h (Figure 1C); however, we noticed an increase in cell number at 120 h, suggesting that cells might adapt to the lack of Ki-67 over time." This is intriguing, but the hypothesis about adaptation should be tested directly by extending the time course in Fig. 1C. Does a subset of the cells indeed begin to proliferative more rapidly after long time periods in Doxycycline + Auxin? Can these late proliferating cells be isolated and analyzed? If so, what happens if the Dox + IAA are removed, and then reintroduced, are the S phase phenotypes lost?

*Reply: This is a very interesting aspect that we have followed up quite intensively. Yes, we can culture these cells for a long period of time without Ki-67: they do grow (albeit slower) but are metabolically different from the parental. We have conducted some detailed characterisations and we are preparing a separate manuscript on this aspect because it would be far too much material to include in this story. However, to respond to the query, and to provide more evidence on our observation and claim at this stage, we are happy to add some initial experiments to this manuscript.*

*We now are showing images of the cell grow in the absence of auxin up to 144 h (Supplementary Figure 1 H). We have also added the data obtained with two derivative cell lines (a reporter of CDK2 activity and a reporter for APC/C^CDH1^ activity) (Supplementary Figure 1 I-J) where we show that the cells without Ki-67 (depleted in an asynchronous population as it is the growth curve presented) sustain an initial block in G1 (low CDK2 and active APC/C^CDH1^) but, after 48 h, the block is overcome and cells start cycling again.*

*We do agree with the referee that this aspect is very important towards the understanding of Ki-67 biology.*

*The modified text reads as follows:*

“To further support this initial observation, we followed the cultures in the presence or absence of auxin for several days by microscopy. This confirmed that indeed the cells with auxin can adapt and start growing, albeit their morphology is different from the ones cultured in the absence of auxin (supplementary Figure 1 H). To obtain more insights on the initial block and the overriding mechanisms, we generated two HCT116Ki-67-AID stable cell lines expressing either the mCherry-CDK2 reporter or the APC/C^CDH1^ reporter [29] [30]. The sensor for CDK2 includes amino acids 994–1087 of human DNA helicase B fused to mCherry, and contains four CDK consensus phosphorylation sites, a nuclear localization signal, and a nuclear export signal. In S phase it has an intermediate activity (localises both to the nucleus and the cytoplasm) while in G0/G1 is inactive (localises only to the nucleus). The APC/C^CDH1^ activity sensor includes the first 110 amino acids of Geminin, the D (destruction) box and the KEN box that are ubiquitinylated leading it to degradation by the APC/C^CDH1^ (Sakaue-Sawano et al., 2008). When APC/C^CDH1^ is not active, GEMININ-mCherry is stable and localises in the nucleus, but when APC/C^CDH1^ is active (before G1/S transition) it degrades GEMININ-mCherry and the signal is lost from the nucleus (Supplementary Figure 1 XX).

Using these cell lines, we could demonstrate that, within 24h of auxin treatment, the cells without Ki-67 block in G1 with low CDK2 activity and active CDH1 but, after 48 h, the blocks is slowly overcome and cells start cycling again (Supplementary Figure 1 I and J).”

2) Conversely, since the previous degron line did display reduced pericentromeric heterochromatin replication upon Ki-67 depletion (we did the FISH and we show that is undereplicated) how different is the new cell line compared to the old regarding this phenotype?

Also, in the old cell line, are the observed effects on the DNA replication proteins (e.g. reduced chromatin association (Fig. 2C-D) or PLA interaction with Mcm3 (Fig. 4G)) not observed?

*Reply: We thank the referee for the comment, and we have asked Dr Takagi’s for his cell line. We have then repeated some key experiments with this cell line.*

*We looked at the effect of replication resumption after Thymidine arrest and we confirmed the same reduction in replicating cells at 4 and 6 hours after release from the block; this is in line with our findings. We have added these new data obtained with the Takagi’s cell line in Supplementary Figure 1 D. The text has also been modified as follows:* “We confirmed the same phenotype using a previously published Ki-67-AID cell line generated using a constitutive expression of OSTR1 (Supplementary Figure 1 D).”

*Therefore 2 different degron cell lines and 2 independent SiRNA oligos have confirmed this phenotype in S phase caused by lack of Ki-67.*

*We have also conducted the PLA experiment in the Takagi’s cell line as the referee requested. The experiments show that an interaction between Ki-67 and MCM3 is also present in the Takagi’s cell line. The new data are presented in the revised Supplementary Figure 3 C, D and the modified text reads as follows: “Similarly, in the PLA assay, we could detect PLA signals between MCM3 and Ki-67 but not upon Ki-67 degradation in both our new cell line and the previously published one (Figure 4 G, H and Supplementary Figure 3 C, D)”.*

3) In Fig. 1D, the time point examined is not mentioned. Indeed, it would be of interest to know how soon the S phase population disappears after auxin treatment.

*Reply: The referee is correct. We added the time of Auxin treatment on the graph.*

4) I believe there is an error in how the diagram for Fig. 1I is drawn. In contrast to Fig. 1E, shouldn't the word "auxin" be placed at time = 0 here?

*Reply: Yes, the referee is correct, we apologise for the mis-labelling. We have fixed the diagram.*

5) The text says that the "short MG132 treatment does not restore Ki-67 levels in cells" (p.11; Fig. 5C), but the drug concentrations and durations used are not mentioned.

*Reply: We added a scheme for the treatment (Figure 5 C) and the time within the text.*

6) A prediction of the HUWE1 data is that treatment with the HUWE1 inhibitor BI8626 will also increase the number of H2AX foci after acute Ki-67 depletion. Is that the case?

*Reply: The referee is correct here, this is indeed the prediction. We have therefore tested this directly by quantifying the number of H2AX foci after acute Ki-67 depletion in presence of the HUWE1 inhibitor BI8626 and added the data in the Revised Figure 5 D. The experiments did show a significant increase of gH2AX foci in this condition although this drug also triggered more DNA damage in the control cells; this probably reflects a baseline function of HUWE1 in DNA replication as previously reported.*

*The modified text reads like this:* “Finally, we confirmed that HUWE1 seems to be the hyperactive ligase triggered by Ki-67 degradation because, by blocking its activity with the specific inhibitor BI8626, we observed both an increase of gH2AX foci (Figure 5 D and E) and TP53 and CHK1 levels (Figure 5 J and Supplementary Figure 4 A, B).

7) Fig. 7. At the time point analyzed, levels of BrdU incorporation in the RO-3306 blocked cells is not significantly reduced after Ki-67 depletion (Fig. 7A-C). This would suggest genome duplication is largely complete. So, do the reduced DNA-FISH signals result from reduced DNA synthesis (i.e. lower copy number) or instead reduced efficiencies of hybridization or detection in this assay? To distinguish these possibilities, quantitative measures of copy number of the loci analyzed should be done. For example, a quantitative PCR or ddPCR analysis could be done.

*Reply: We thank the referee for making us reflect on this point. We did not measure the levels of BrdU incorporation but only the percentage of cells that incorporated BrdU. I am also not really confident that measuring the bulk of BrdU incorporation would be an accurate measure of the levels of DNA replication: the visualisation of BrdU requires quite a harsh treatment, and I would not be confident in using it as a quantitative measure of replication aside from being a very rough indicator. The data presented only suggest that the majority of cells complete replication because they are BrdU positive.*

*Regarding the reduced signals:*

1) *The referee can be correct for the analyses of the centromeric satellites as we are comparing signals coming from different cells. Unfortunately, the PCR analyses is not feasible as these are highly repetitive regions. However, our data are in agreement with the ones recently published by the Van Steelsen’s group where they observed delay in replication timing specifically at (peri-)centromeres.*

*From a technical point of view, it needs to be noted that, without Ki-67, the area occupied by the probe (pericentromeric) is larger and this indicates a more relaxed chromatin arrangement that should be more favourable for probe accessibility, and you could expect a stronger hybridization signal. However, the intensity is lower.*

*We have added some discussion points on this aspect.*

2) *For the single copy probe, we can be more confident because we are comparing the relative intensity of sister chromatids; this eliminates the variability of denaturation /hybridization efficiency between nuclei.*

8) The comparison of Ki-67 expression levels with other genes is complicated by the fact that Ki-67 expression is highly correlated with cell proliferation. Therefore, the significance of the comparisons with the IFIT1 and STAT2 expression levels should be tested by asking whether other proliferation-associated gene expression signatures display similar correlations.

*Reply: We thank the referee for this comment. We do agree that bioinformatically this is a complex relationship to dissect. In fact, most of the classic proliferation markers (PCNA, MCM) do correlate with Ki-67 and they do negatively correlate with an interferon response. The one that does not is MYC, which, per se, is already interesting.*

*Therefore, to answer this point we have resourced to an experimental approach. The key here is being able to separate proliferation from Ki-67 levels and then analyse the interferon response. During this study, we collected 3 HCT116 cell lines that are growing and adapted to different levels of Ki-67. The new cell line we have generated (highest level), the Takagi’s cell line (intermediate level because the constitutive expression of OSTR1 causes some base line degradation) and the cell line we have obtained by maintaining our cells in presence of Dox and auxin (Ki-67 almost undetectable). The western blot analyses of these cell lines (Supplementary Figure 5 F, G) shows that the cell lines have the same levels of PCNA but very different levels of Ki-67. We therefore tested the strength of the interferon response using the reporter system described in Figure 3. The data obtained show that there is a negative correlation between the interferon response strength and the levels of Ki-67 (Supplementary figure 5 H, I). This therefore uncouples proliferation form Ki-67 and links the interferon response to Ki-67 levels and not proliferation potential.*

*The modified text reads as follows:*

Interestingly, the two genes show a strong negative correlation among several datasets, but no correlation is found with MYC, an oncogene linked to proliferation (Supplementary Figure 5 E). However, in order to uncouple proliferation and Ki-67 levels, we used 3 different HCT116 cell lines that present different amount of Ki-67: 1) the cell line we have generated in this paper that expresses a high level of Ki-67, 2) the previously published Ki-67-AID cell line that expresses a lower level of Ki-67 because the OSTR1 is constitutively expressed under the CMV promoter and 3) the cell line we have generated and maintained always in Dox and Auxin that expresses almost negligible levels of Ki-67 (Supplementary Figure 5 F, G). All these cell lines proliferate and have the same amount of another classic proliferation marker, PCNA (Supplementary Figure 5 F, G). Using these cell lines, we then transfected the interferon reporter system described before (Figure 3 C) and measured the luciferase intensity. The data show that Ki-67 levels negatively correlate with the strength of the interferon response, independently of other proliferation markers (Supplementary Figure 5 H, I).

9) Places for text proofreading:

p.3, line 57: the phrase "and positioning" seems misplaced, I think it was meant to follow the words "compromised heterochromatin maintenance".

*Reply: Thanks, we have fix this.*

p.10, line 8: "not relying on antibodies of solubilisation problems". I think "of" should be "or" here.

*Reply: Yes, we corrected this.*

**Second round of review**

**Reviewer 2**

The authors have responded thoroughly to the reviewers’ comments. I think there are only minor changes required prior to acceptance.

I like the approach comparing the IFN gene transcriptional response in the cell lines with different amounts of Ki-67. I realize that all three lines do grow, but do we know whether the doubling times of these three lines are the same? That is, it could be the rate of proliferation that is the key factor rather than the Ki-67 protein levels?

Given that Supplemental Figure 1D now includes analysis of the HCT116:Ki- 67-AIDCMV:OSTR1
cells from the Takagi lab, the legend must indicate that the Tet-On version of the cells are used elsewhere in the figures.

Finally, there are a couple of places in the Abstract where the wording should be adjusted to better differentiate this work from previous studies.
1. Since this is not the first degron used to study Ki-67, I would add these words to the beginning of the Results section of the Abstract: “Here we have used a doxycycline-inducible E3 ligase together with an auxin-inducible degron tag (AID) to achieve a rapid…”

2. “However, a role for Ki-67 in regulating cell cycle progression has never been reported.” This sentence will confuse readers who are familiar with the many prominent papers about Ki-67’s roles in shaping chromosomes during mitosis and ensuring proper separation of nuclear and cytoplasmic material during mitotic exit. I would replace this sentence with something like “However, how the different roles for Ki-67 across the cell cycle are regulated and coordinated remain poorly understood”.

**Reviewer 3**

I was asked to comment on the revised manuscript and the rebuttal letter in response to reviewer one. The authors have generated several HCT116 knockin cell lines, including AID, Turbo-ID, and cell cycle-dependent degron cells, to study the function of Ki-67 and its role in DNA replication. Compared with the relevant model systems and previous publications, the new cell lines provide specific value but require stringent characterization if they want to focus more on resources. However, if the primary purpose is to reveal the role of Ki-67 on DNA replication and underlying molecular mechanisms, it is still in the early phase and lacks sufficient mechanism support. To me, the revised version falls in a piece combining both directions.

The main argument between reviewer one and the authors is the necessity of generating the new AID cell lines and significant phenotype upon auxin-induced degradation. Compared to other crude knockout strategies, including siRNA, CRISPR KO, or Cre/LoxP systems, the AID system provides an acute and reversible protein degradation to study the direct protein function. I have to say it is unfair to accuse the integrity and reliability of hush words without solid evidence. However, to support the significant comments raised by the authors, independently derived knockin clones and rescue experiments are required. Unfortunately, the authors heavily relied on one cell line and tried to address the rebuttal to another historical cell line derived by other researchers. As both parties may already realize, the genome-editing approach for AID knockin would cause possible unwanted effects in cells. Therefore, it is very important to unbiasedly generate multiple clones to support the main conclusion, which helps to exclude the Ki-67 unrelated effect. Also, washout treatment is another potent strategy to further identify Ki-67 function. The authors also claimed this is the first time to link Ki-67's role with DNA replication, which is also an overstatement. How Ki-67 regulates the functional network in DNA replication was not indeed clearly illustrated in this manuscript.

Minor comments
1. Figure design and quality control are not showing as excellent quality. I observed multiple different fonts and word styles in figures.
2. There are no Dox+/- only groups as negative controls for significant experiments.
3. Lack of QC of degradation efficiency at single cell level. WB is a relative quantification of protein level. However, residual protein at chromatin cannot be ignored in the AID system.
4. Given the cell cycle distribution in bulk population, single cell-based profiling may help reveal the function of Ki-67 and dosage-dependent effect.

**Authors Response**

**Point-by-point responses to the reviewers’ comments:**

Reviewer #2

The authors have responded thoroughly to the reviewers’ comments. I think there are only minor changes required prior to acceptance.

*Reply: We thank the referee for recognising that “The authors have responded thoroughly to the reviewers’ comments. I think there are only minor changes required prior to acceptance.”*

I like the approach comparing the IFN gene transcriptional response in the cell lines with different amounts of Ki-67.

*Reply: We thank the referee for the appreciation.*

I realize that all three lines do grow, but do we know whether the doubling times of these three lines are the same?

*Reply: The doubling times are not the same and we added them to the figure (Supplementary Figure 5 I).*

That is, it could be the rate of proliferation that is the key factor rather than the Ki-67 protein levels?

*Reply: We were quite intrigued by this comment and suggestion therefore we have plotted the doubling time and interferon response (added in Supplementary Figure 5 J). There is indeed a positive correlation between interferon response and doubling time. This is interesting but also strongly suggests that, in this cell line, the doubling time is positively correlated to the level of Ki-67, something that was unknown before. Thanks for the suggestion. We have incorporated this in the text. Now the test reads as follow:*

*Interestingly, the strength of the interferon response also negatively correlates with the doubling time of the cell lines, which in turns indicate that Ki-67 levels positive correlate with the doubling time at least in HCT116 cells (Supplementary Figure 5 J).*

Given that Supplemental Figure 1D now includes analysis of the HCT116:Ki- 67-AIDCMV:OSTR1
cells from the Takagi lab, the legend must indicate that the Tet-On version of the cells are used elsewhere in the figures.

*Reply: Yes, good point. We added the following statement in the text: Therefore for the remaining experiments, unless specifically indicated, the Ki-67-AID cell line refers to the Ki-67-AID Dox:OSTR1.*

Finally, there are a couple of places in the Abstract where the wording should be adjusted to better differentiate this work from previous studies.
1. Since this is not the first degron used to study Ki-67, I would add these words to the beginning of the Results section of the Abstract: “Here we have used a doxycycline-inducible E3 ligase together with an auxin-inducible degron tag (AID) to achieve a rapid…”

2. “However, a role for Ki-67 in regulating cell cycle progression has never been reported.” This sentence will confuse readers who are familiar with the many prominent papers about Ki-67’s roles in shaping chromosomes during mitosis and ensuring proper separation of nuclear and cytoplasmic material during mitotic exit. I would replace this sentence with something like “However, how the different roles for Ki-67 across the cell cycle are regulated and coordinated remain poorly understood”.

*Reply: Yes, we agree with the suggestions made and we have changed the abstract using those statements.*

Reviewer #3

I was asked to comment on the revised manuscript and the rebuttal letter in response to reviewer one. The authors have generated several HCT116 knockin cell lines, including AID, Turbo-ID, and cell cycle-dependent degron cells, to study the function of Ki-67 and its role in DNA replication. Compared with the relevant model systems and previous publications, the new cell lines provide specific value but require stringent characterization if they want to focus more on resources. However, if the primary purpose is to reveal the role of Ki-67 on DNA replication and underlying molecular mechanisms, it is still in the early phase and lacks sufficient mechanism support. To me, the revised version falls in a piece combining both directions.

The main argument between reviewer one and the authors is the necessity of generating the new AID cell lines and significant phenotype upon auxin-induced degradation. Compared to other crude knockout strategies, including siRNA, CRISPR KO, or Cre/LoxP systems, the AID system provides an acute and reversible protein degradation to study the direct protein function. I have to say it is unfair to accuse the integrity and reliability of hush words without solid evidence. However, to support the significant comments raised by the authors, independently derived knockin clones and rescue experiments are required. Unfortunately, the authors heavily relied on one cell line and tried to address the rebuttal to another historical cell line derived by other researchers.

*Reply: We are well aware that there was a lot of material in the manuscript and that some parts must have escaped the referee’s attention. We were indeed very careful with our analyses, and this is the reason why the two major points of the work 1) DNA replication delay and 2) the interferon response were addressed with several independent methods.*

*Point 1) was also addressed using an RNAi approach with 2 different and validated oligos (Supplementary Figure 1 F of the previous manuscript).*

*Point 2) was also addressed not only using 2 different validated oligos but also 6 different cell lines.*

*The extra validation with the previously published cell line has been an editorial request, this is why it has also been added. I think that this is the most rigorous validation of any AID cell line ever generated in the literature, not only for Ki-67 but for several other AID cellular models.*

As both parties may already realize, the genome-editing approach for AID knockin would cause possible unwanted effects in cells. Therefore, it is very important to unbiasedly generate multiple clones to support the main conclusion, which helps to exclude the Ki-67 unrelated effect.

*Reply: The referee is correct, you need several independent clones and, of course, we did these tests before embarking on the study. As requested, we have added the data from them in Supplementary Figure 1 I, J). We have added the data from 2 additional independent clones as examples (clone #2 and clone #3). I think this should be sufficient to satisfy the referee also considering all the extensive validation we have conducted by several other methods (see response above).*

*The modified text now reads as follows:*

*We confirmed the same phenotype using a previously published Ki-67-AID cell line generated using a constitutive expression of OSTR1 [29] (Supplementary Figure 1 D) and two additional independent clones generated with our method (Supplementary Figure 1 I and J). Therefore for the remaining experiments, unless specifically indicated, the Ki-67-AID cell line refers to the Ki-67-AIDDox:OSTR1.*

Also, washout treatment is another potent strategy to further identify Ki-67 function.

*Reply: We have added a wash out treatment for the growth curve of cells kept in auxin for a prolonged period of time. As can be seen, Ki-67 levels are restored upon auxin wash out and the cell growth is restored (supplementary Figure 1 O, P). Now the reads as follows:*

*To further confirm that the growth delay in presence of auxin was really linked to Ki-67 levels, we used the cell line that we kept growing in auxin for months (Ki-67-AID + auxin (prolong treatment) and removed the auxin to assess the growth ability, doubling time and Ki-67 levels restoration. These experiments revealed that the removal of auxin (wash out treatment) (Ki67-AIDauxin_wash_out line) restores the original levels of Ki-67 (Supplementary Figure 1 P), and that the growth curve is similar to the original cell line (before the addition of auxin) (Supplementary Figure 1 O).*

The authors also claimed this is the first time to link Ki-67's role with DNA replication, which is also an overstatement. How Ki-67 regulates the functional network in DNA replication was not indeed clearly illustrated in this manuscript.

*Reply: We do disagree with the statement made by the referee, but we do not want to enter in a semantic argument, therefore we have modified the statement.*

Minor comments
1. Figure design and quality control are not showing as excellent quality. I observed multiple different fonts and word styles in figures.

*Reply: All figures were submitted as PDF 1200 dpi so it is not possible to have a higher resolution than this. The figures look very high resolution on our side. Maybe the system has changed them for the referee. I do not know. The fonts are all Arial and the size conforms to the graph/image type. We have double-checked all of them.*

2. There are no Dox+/- only groups as negative controls for significant experiments.

*Reply: Maybe the referee has not noticed that there are indeed several experiments where the dox alone was used (e.g the important replication machinery loading Figure 2C). As we show in the figures, the Dox alone induces the OSTR1 which is leaky (this point has been heavily discussed in the paper and in the previous rebuttal letter), thus causing already Ki-67 degradation (supplementary Figure 1 C). For this reason, some of the experiments using just dox as a control are meaningless as they will produce an effect similar to the one observed with decreased Ki-67 levels (Takagi’s cell line for example). As we have clearly demonstrated, the interferon response and the doubling time are some of those parameters. Where this control makes sense, it was added.*

*We have also added in the revision, the replication delay with the Dox control for the two extra independent clones used (Suppletory Figure 1 I, J).*

3. Lack of QC of degradation efficiency at single cell level. WB is a relative quantification of protein level. However, residual protein at chromatin cannot be ignored in the AID system.

*Reply: We have also added a quantification of cells where Ki-67 was undetectable by microscopy in Supplementary Figure 1 D and E to support the western blot analyses. Moreover, it must be noted that the majority of our assays are single-cell based (see also response to the point below).*

4. Given the cell cycle distribution in bulk population, single cell-based profiling may help reveal the function of Ki-67 and dosage-dependent effect.

*Reply: The majority of our assays are indeed single-cell based. These are:*

*1) replication timing and progression (Figure 1 E-K)*

*2) Comet assay and STING KO effect (Figure F-J)*

*3) PLAs with replication machinery, Ki-67 foci distribution and BrdU incorporation (Figure 4 J-K and Supplementary Figure 3 C-F)*

*4) gH2AX foci and response to Huwe1 inhibition (Figure 5 D, E)*

*6) Replication defects and cohesion (Figure 7 B-F)*

*7) Cell cycle arrest and restart (CDK2 and APC/C) (Supplementary Figure 1 M, N and Supplementary Figure 4 C, D)*

*The correlation between Ki-67 levels and interferon response was experimentally demonstrated (Supplementary Figure 5 F-J).*

*Therefore, for each single important point we have made, there is a single-cell approach in the manuscript. Only the RNA-seq and phosphoproteomics are bulk analyses. Single cells RNA-seq would be really going beyond the scope of this study and single cells phosphoproteomic is not an approach available at the moment.*

**Third round of review**

**Reviewer 2**

The authors have responded thoroughly to the reviewers' comments, and I think this important study is now ready for publication.

**Reviewer 3**

I am okay with scientific rebuttal for the questions related more clones, independent loss of function and controls.
However, I am not satisfied with the figure presentation and relative low quality as I mentioned in the last review. They claimed that they made all fronts and sizes of words in the same style, which is still not consistent and clear. All kinds of font and word size are flowing crossing the entire figures. Of course, this is not directly pointed to science merit.

**Authors Response**

**Point-by-point responses to the reviewers’ comments:**

Reviewer #3

I am okay with scientific rebuttal for the questions related more clones, independent loss of function and controls.
However, I am not satisfied with the figure presentation and relative low quality as I mentioned in the last review. They claimed that they made all fronts and sizes of words in the same style, which is still not consistent and clear. All kinds of font and word size are flowing crossing the entire figures. Of course, this is not directly pointed to science merit.

*Reply: We have tried out best in standardise the fonts (all ariel) and size (compatibly with the panels of the figures). What we have uploaded on the portal are 1200 dpi PDFs (aside form one figure that would be too big for the journal at this resolution): I am not sure we can improve on this. On our side they look excellent quality and resolution.
I hope you will agree.*
